# Common-Garden Transcriptomes Distinguish Leaf-Form-Associated from Site-Associated Expression in *Zygophyllum xanthoxylum*

**DOI:** 10.3390/ijms27198537

**Published:** 2026-09-24

**Authors:** Xiaoyang Wang, Wenjing Shen, Xingshun Wang, Duanyang Yuan, Qin Lu, Zhentao Ren, Ming Zhu

**Affiliations:** 1 Biological and Medical Metrology Laboratory, Beijing Institute of Metrology, Beijing 100029, China; wangxiaoyanghu@gmail.com; 2 College of Life and Environmental Sciences, Minzu University of China, Beijing 100081, China; 3 Yunnan Open University, Kunming 650223, China; shenwenjing@ynou.edu.cn (W.S.); 11653014@mail.sustech.edu.cn (X.W.); yuanduanyang@ynou.edu.cn (D.Y.); 4 Institute of Highland Forest Science, Chinese Academy of Forestry, Kunming 650224, China; lqin@caf.ac.cn; 5 Institute of Food and Nutrition Development, Ministry of Agriculture and Rural Affairs, Beijing 100081, China; renzhentao@caas.cn

**Keywords:** *Zygophyllum xanthoxylum*, leaf shape, de novo transcriptome, light-harvesting complex, KNOX, desert plant

## Abstract

*Zygophyllum xanthoxylum* (Bunge) Maxim. is a dominant, deep-rooted shrub of the temperate deserts of north-western China whose leaflets range from comparatively flat and broad to slender and terete, but the molecular basis of this variation has rarely been reported. We compared three accessions from contrasting desert habitats—one flat-leaved, from a shaded high-altitude valley, and two rod-leaved, from open full-sun sites. The accessions differed in the leaflet blade but not in the petiole, the flat-leaved accession bearing shorter and much wider leaflets with a width-to-length ratio almost four times greater. Leaf transcriptomes were assembled, and pairwise comparison identified several thousand differentially expressed genes. The leaf-form signal was defined as the genes that changed concordantly in both flat-versus-rod comparisons, placing the flat-leaved accession at one extreme of the series. This set contains a reciprocal leaf-development module: the YABBY lamina gene *YAB2*, the auxin/polarity factors *ARF3* and *ARF18* and the expansin *EXPA1* are higher in the flat-leaved accession, whereas the KNOX gene *KNAT6*, the class II TCP gene *TCP12*, *ARF6*, *EXPA4* and *ATHB7* are higher in both rod-leaved accessions. Photosynthesis genes are significantly concentrated in the expression clusters that peak in the flat-leaved accession, with light-harvesting antenna, photosystem II and Calvin-cycle transcripts highest there. The chlorophyll *a*-*b* binding protein *CAB* and the cytosolic phosphoglycerate kinase *PGK* are the most strongly flat-biased transcripts, and in each case, the bias holds for the gene family as a whole; quantitative RT-PCR confirmed the sequencing-based estimates. Flat- versus rod-leaved differentiation in *Z. xanthoxylum* is therefore accompanied by constitutive, coordinated differences in leaf-development and light-harvesting gene expression, *CAB* and *PGK* are proposed as candidate expression markers of the flat-leaved form.

## 1. Introduction

Leaves are the primary organs of light capture and gas exchange, and their shape is among the most plastic and adaptively important traits in plants [1,2]. In arid environments, leaf size and geometry sit at the centre of a well-known trade-off: a broad, flat blade intercepts light efficiently and supports high photosynthetic rates [3] but exposes a large surface to evaporative demand and heat load [4,5]; a narrow, cylindrical or terete leaf sacrifices light-harvesting area in exchange for a smaller surface-to-volume ratio, reduced transpiration and better tolerance of high irradiance and drought [6]. Desert shrubs frequently sit at the rod-leaved end of this continuum [7], and shifts towards reduced, terete or scale-like leaves recur in lineages that have colonised hot, dry, high-light habitats [8].

*Zygophyllum xanthoxylum* (Bunge) Maxim. (Zygophyllaceae, subfamily Zygophylloideae) is a deep-rooted, succulent-leaved shrub that dominates gravel and sandy deserts across arid north-western China and neighbouring Central Asia [9]. It is a keystone species for sand fixation and desertification control, as well as a valuable browse and medicinal plant. Its ability to accumulate Na^+^, compartmentalise it in the vacuole and thereby maintain a favourable water status under severe stress has also made it a model for research on salt and drought tolerance [10,11]. Within the species, individuals from different habitats vary conspicuously in leaf form, from comparatively flat and broad to slender and rod-like. This intraspecific variation offers a natural experiment: plants that share a species-level genetic background but originate from contrasting environments can be compared to identify the genes whose expression accompanies—and may underlie—the divergence in leaf shape and in the physiological strategies that go with it [12,13].

The genetic control of leaf shape is understood in broad outline from model species [14]. Flattening of a blade depends on the establishment of adaxial–abaxial polarity: class III HD-ZIP transcription factors such as *REVOLUTA* and *ATHB15/CORONA* specify adaxial identity [15,16], KANADI and YABBY factors specify abaxial identity and drive lamina outgrowth [17] and juxtaposition of the two domains is required for a flat blade to form. Superimposed on this framework, class I KNOTTED1-like homeobox (KNOX) genes maintain the indeterminate, meristematic state and must be switched off in leaf primordia for a determinate, flattened leaf to develop [18,19]; their ectopic or prolonged expression produces lobed, radialised or otherwise altered leaves. Auxin patterns the leaf margin and reinforces polarity through AUXIN RESPONSE FACTORs such as ARF3/ETTIN [20]. Final blade size is then set by the balance between class II TCP factors, which restrain proliferation [21], and GROWTH-REGULATING FACTORs together with cell-wall-loosening expansins, which drive proliferation and expansion [22,23]. How this conserved toolkit is deployed to produce the flat-versus-rod contrast seen within a desert shrub such as *Z. xanthoxylum* has not been examined [24].

Here we compare three accessions of *Z. xanthoxylum*, one flat-leaved and two rod-leaved, raised together from seed under uniform common-garden conditions and profiled by de novo leaf transcriptome sequencing. Because the accessions differ in the severity of their source habitat as well as in leaf form, the two effects are superimposed in any single pairwise comparison; we therefore partitioned the differentially expressed genes into a leaf-form axis and two site axes before interpreting them. Our aims were (i) to quantify the morphological divergence among the accessions; (ii) to describe the global transcriptional differences among them; (iii) to resolve the leaf-form axis from the site axes and identify the leaf-development and photosynthesis genes associated with each; and (iv) to nominate candidate expression markers of the flat-leaved form.

## 2. Results

### 2.1. The Three Accessions Come from Contrasting Habitats and Differ in Seed Size and Packing

The three source sites span a clear environmental gradient (Figure 1A,B; Table 1). Guide lies highest, at about 2660 m, where the plants grow shaded beneath a plantation canopy and therefore in comparatively low light; the two Xinjiang sites are lower and fully sun-exposed. Continentality runs counter to altitude, the low, open Xinjiang sites swinging through annual temperature ranges of roughly 37 °C at Korla and 33 °C at Wensu against 24 °C at the higher Qinghai site. Guide therefore occupies the high, shaded, cool-summer end of the gradient and Korla the low, bright, extreme-continental end, with Wensu intermediate in temperature range but, like Korla, fully sun-exposed and arid.

Seed morphology tracked this contrast (Figure 1C–E). Thousand-grain weight was essentially identical across accessions, close to 14 g in each, yet the seeds were not built the same way. Guide produced the largest seeds, 8.63 × 3.36 mm on average against 7.66 × 2.83 mm in Korla and 7.59 × 3.04 mm in Wensu, and correspondingly the largest projected area and estimated volume, the latter spanning a 1.6-fold range. Because a similar mass was packed into a larger volume, Guide seeds were the least dense and Korla the densest. The flat-leaved, high-altitude accession thus makes larger, less dense seeds and the rod-leaved, arid accessions smaller, denser ones. Projected area, volume and density are geometric estimates computed from the mean axis lengths and are used here as descriptors of seed shape and packing.

### 2.2. The Leaflet Blade, but Not the Petiole, Distinguishes the Flat- from the Rod-Leaved Accessions Under Common-Garden Conditions

Grown side by side from seed, the three accessions looked broadly similar in the first week, but by 30 days, the difference in leaf form was unmistakable: Guide bore flat, broad leaflets, whereas Korla and Wensu produced slender, terete leaflets in both adaxial and abaxial views (Figure 2A). Morphometrics localised the contrast to the blade. The petiole did not differ among accessions in length, width or width-to-length ratio (Figure 2B). The leaflet blade differed sharply (Figure 2C): relative to the two rod-leaved accessions, Guide had shorter leaflets, 2.15 cm against 3.90 and 4.30 cm, but much wider ones, 0.55 cm against 0.27 and 0.30 cm, so that its width-to-length ratio of 25.6% was 3.7-fold higher than the 6.9% and 7.0% recorded for Korla and Wensu, respectively. The two rod-leaved accessions did not differ from each other. The central phenotype of this study is therefore a flat, broad blade in Guide against narrow, rod-like blades in Korla and Wensu, with the petiole conserved; because all plants were raised under identical conditions, this difference is constitutive.

**Figure 2:**
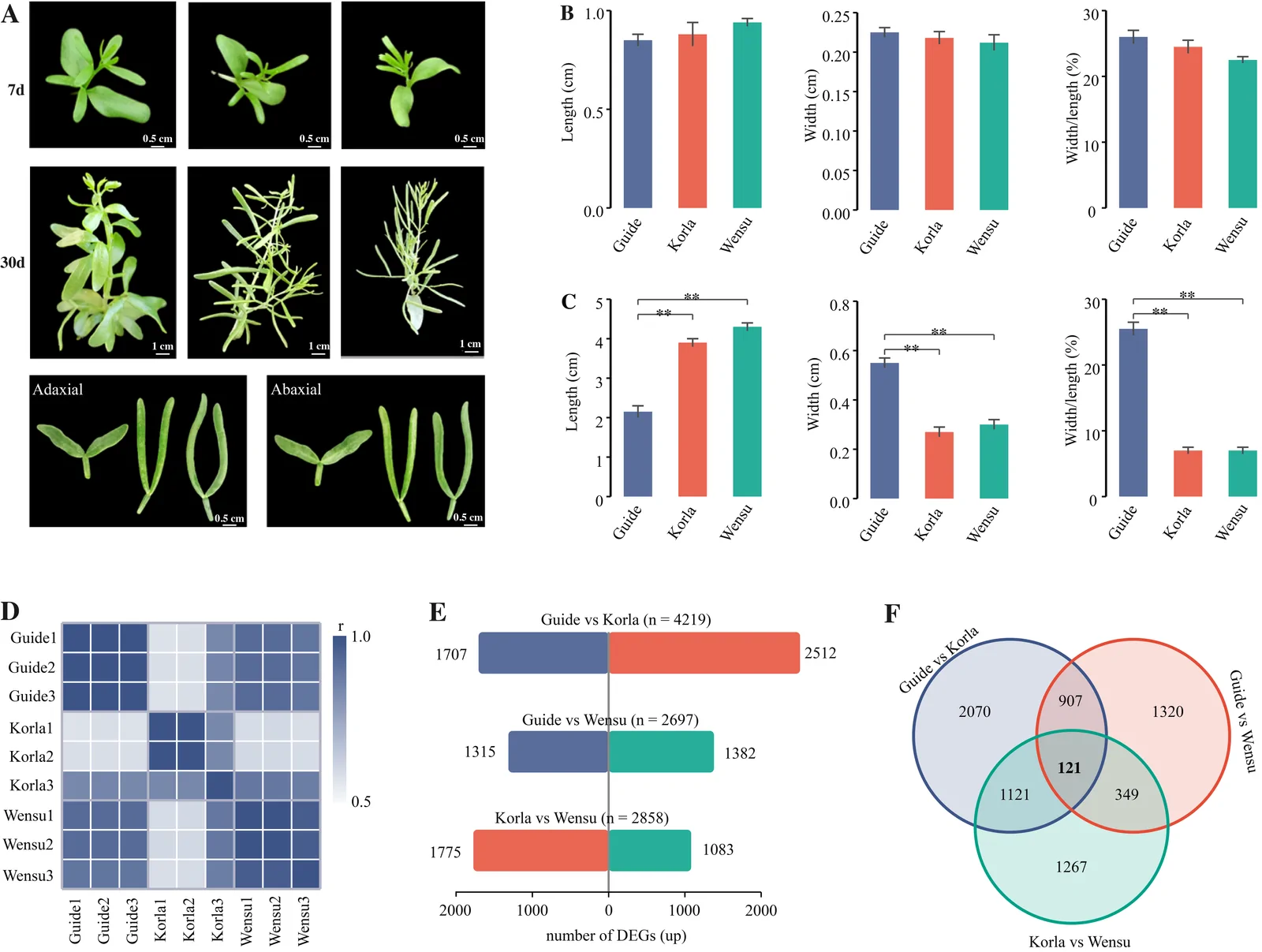
Seedling morphology under common-garden conditions and the transcriptome landscape. (**A**) Seedling phenotype 7 and 30 days after transplanting and adaxial and abaxial views of representative leaves, showing the flat, broad leaflets of Guide against the terete leaflets of Korla and Wensu. Morphometric data in (**B**,**C**) are from ten independent plants per accession, one fully expanded leaflet from the middle of the shoot per plant, so that the plant and not the leaf is the statistical unit (*n* = 10 plants per accession). Bars in (**B**,**C**) are accession means and error bars the standard deviation of those ten plants. (**B**) Petiole length, width and width-to-length ratio: no significant differences among accessions (one-way ANOVA, *p* > 0.05; ns, not significant). (**C**) Leaflet-blade length, width and width-to-length ratio: Guide has significantly shorter and wider leaflets and a 3.7-fold higher width-to-length ratio than Korla and Wensu, which do not differ from each other (Dunnett’s test against Guide; ** *p* < 0.01). (**D**) Pairwise Pearson correlation of the nine libraries computed across all genes from TPM values; colour, correlation coefficient. Replicates correlate closely within each accession; between accessions, Guide and Wensu are the most similar pair, and Korla is the most divergent. (**E**) Numbers of DEGs per comparison (|log_2_ fold change| ≥ 2, adjusted *p* < 0.01, detected in every replicate); bars extend left for genes higher in the first accession and right for genes higher in the second. (**F**) Venn diagram of the three DEG sets; 121 genes are differentially expressed in all three comparisons.

### 2.3. A Complete De Novo Reference and Reproducible Libraries

The nine leaf libraries yielded 42.1–51.9 million raw and 32.7–50.9 million clean paired-end reads of uniformly high quality (Table S1; Figure S1). Pooled assembly and Corset clustering produced 118,214 genes spanning 163.7 Mb, with a mean length of 1385 bp and an N50 of 1993 bp, just over half of them at least 1 kb long (Table S2). BUSCO recovered 423 of the 425 viridiplantae_odb10 single-copy orthologues as complete, the high proportion of duplicated orthologues being expected for a clustered transcriptome, which indicates an essentially complete representation of the gene space. Functional annotation assigned 78.9% of genes in at least one database (Table S2).

### 2.4. The Korla Accession Is the Most Divergent Transcriptome-Wide, and the DEGs Are Dominated by Regulatory, Signalling and Transport Functions

Replicate libraries were reproducible, and between accessions, Guide and Wensu formed the most similar pair, while Korla was clearly set apart from both (Figure 2D and Figure S2). The numbers of differentially expressed genes agreed: Guide versus Wensu gave the fewest DEGs (2697), Korla versus Wensu an intermediate number (2858) and Guide versus Korla the most (4219), with the direction of change in each comparison shown in Figure 2E. Across the three comparisons 7155 distinct genes were differentially expressed, 92.0% of them carrying a functional annotation. Most were specific to a single comparison, while 907, 1121 and 349 genes were shared by exactly two comparisons, and a core of 121 was differentially expressed in all three (Figure 2F; per-comparison volcano plots are shown in Figure S3). Global transcriptional divergence is therefore governed primarily by the distinctiveness of the extreme-habitat Korla accession rather than by leaf form, a point that shapes the analysis that follows.

The DEGs themselves are dominated by regulatory and transport functions. Assigning them to broad functional categories gave a consistent picture across the three comparisons (Figure 3A). Protein kinase and signalling genes formed the largest category in every comparison, followed by transcription factors, transport, stress and defence, hormone signalling, cell wall, cuticle and wax, secondary metabolism and photosynthesis; the ranking was the same whichever pair of accessions was compared. Among the 419 DEG-encoded transcription factors (Figure 3B), WRKY was the largest family, ahead of bHLH, AP2/ERF, NAC and MYB. GO and KEGG enrichment reinforced this picture (Tables S3 and S4; Figures S4–S8): terms describing responses to chemical, endogenous, hormonal and external stimuli were enriched in all three comparisons, secondary-metabolite biosynthesis and general metabolic pathways were the two leading KEGG pathways throughout and photosynthesis-related pathways appeared only in the comparisons that involved the flat-leaved Guide accession.

**Figure 3:**
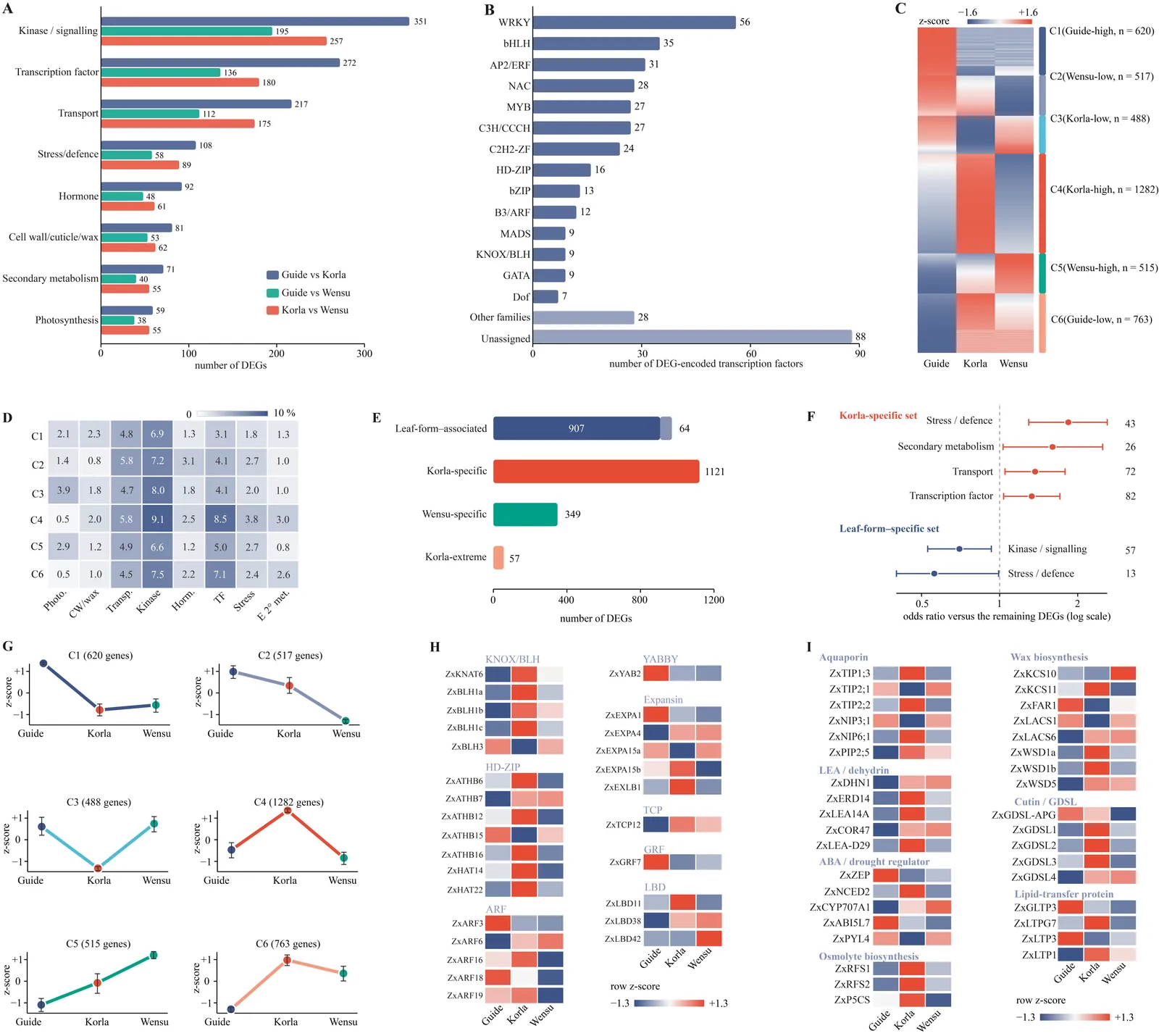
From the global functional landscape to the leaf-form axis and candidate genes. Functional categories and transcription-factor families are homology-based and may overlap. (**A**) Number of DEGs per functional category in the three comparisons. (**B**) Transcription-factor families among the 419 DEG-encoded transcription factors (union of the three comparisons). (**C**) Heat map of the 4185 DEGs for which normalised expression estimates were available for all three accessions, k-means clustered (k = 6) on the row-scaled mean expression per accession; left colour bar, clusters. (**D**) Functional composition of each cluster: both the printed value and the colour are the percentage of the cluster’s genes assigned to that category, on the single scale shown at the right (categories are not exhaustive, so rows do not sum to 100%). (**E**) Partition of the DEGs along the axes defined in Section 4.6. Left, size of each set; the leaf-form–associated bar is subdivided into leaf-form–specific (dark, 907) and leaf-form–graded (light, 64) genes, of which 363 are higher in the flat-leaved Guide and 608 in both rod-leaved accessions, while the Korla-specific set comprises 793 genes higher and 328 lower in Korla. Right, functional categories that are over- or under-represented in each set relative to all remaining DEGs (Fisher’s exact test, two-sided): points are odds ratios on a log scale, bars are 95% confidence intervals, the dashed line marks an odds ratio of 1 and n is the number of genes of that category in the set. (**F**) Mean expression trajectory, size and marker genes of each cluster; points are the cluster mean row z-score per accession, error bars represent the SD across the genes of the cluster and the dotted line marks z = 0. The row z-score standardises the three accession means of each gene to a mean of 0 and a standard deviation of 1, so the panels describe the shape of each expression profile rather than its absolute level; the underlying values and the representative marker genes of each cluster are given in Table S8. (**G**,**H**) Row-scaled (z-score) heat maps of representative candidate DEGs grouped by family: (**G**) leaf-development and meristem genes; (**H**) cuticle, water-relations and drought genes. The colour scale shared by the two panels is shown between them and saturates at a row z-score of ±1.3. (**I**) aquaporin and wax biosynthesis genes. The colour scale shared by the two panels is shown between them and saturates at a row z-score of ±1.3. Gene abbreviations, full names, identifiers and classifications are in Table S7.

### 2.5. The Transcriptional Variation Resolves into One Leaf-Form Axis, Two Site Axes and Six Co-Expression Clusters

Because Korla dominates the global signal, the leaf-form contrast cannot be read directly from any single pairwise comparison. We therefore partitioned the DEGs (Figure 3E). The leaf-form–associated set—genes differentially expressed in the same direction in both Guide versus Korla and Guide versus Wensu, so that the flat-leaved accession lies at one extreme relative to both rod-leaved accessions—contained 971 genes, of which 363 were higher in the flat-leaved Guide and 608 higher in both rod-leaved accessions. Most of these, 907 genes, are leaf-form–specific, showing no residual difference between Korla and Wensu; the remaining 64 are leaf-form–graded, keeping the same flat-versus-rod direction, while the two rod-leaved accessions also differ in magnitude. The Korla-specific set contained 1121 genes (793 higher and 328 lower in Korla) and the Wensu-specific set 349 genes, while a further 57 genes were Korla-extreme, differentially expressed in all three comparisons with Guide intermediate between the two rod-leaved accessions.

The two large sets differ in functional content in a way that confirms they capture distinct biology (Figure 3E). Relative to the remaining DEGs, the Korla-specific set was significantly enriched for stress and defence, transport amd transcription-factor and secondary-metabolism genes, whereas the leaf-form–specific genes showed the opposite tendency and were significantly depleted of stress and defence genes and of kinase and signalling genes. The genes that track leaf form are therefore not a stress signature, and the genes that set the harshest site apart are not a leaf-shape signature.

An unsupervised view of the same data agrees with this partition. The 4185 DEGs for which normalised expression estimates were available for all three accessions resolved into six co-expression clusters (Figure 3C,F). Two clusters peaked in the flat-leaved Guide accession—C1 (Guide-high, *n* = 620) and C2 (high in Guide, low in Wensu, *n* = 517)—one was Korla-low with similar expression in Guide and Wensu (C3, *n* = 488), and three peaked in one or both rod-leaved accessions: C4 (Korla-high, *n* = 1282, the largest cluster), C5 (Wensu-high, *n* = 515) and C6 (Guide-low, high in both rod-leaved accessions, *n* = 763).

Functional composition differed markedly among clusters (Figure 3D). Photosynthesis genes were most frequent in C3 and C1 and rarest in C4 and C6, whereas transcription factors, kinases, stress and secondary-metabolism genes were concentrated in C4 and C6. The bias was significant for the curated sets: half of the photosynthesis DEGs lay in C1 and C3, which together hold only about a quarter of the clustered DEGs, and they were correspondingly depleted from the rod- and Korla-high clusters C4 and C6. Cuticle, water-relations, drought, aquaporin and wax biosynthesis genes showed no such bias, although several individual members peaked strongly in Korla (Figure 3H,I). Marker genes reinforced these identities, C1 and C3 carrying light-harvesting and Calvin-cycle components and C4 and C6 defence, protease and isoprenoid enzymes (Table S8).

### 2.6. A Reciprocal Leaf-Development Module Tracks the Flat-Versus-Rod Contrast

Mining the leaf-development families produced a coherent, reciprocal module in which lamina-associated and rod-associated genes moved in opposite directions (Figure 3G and Figure 4A; Table S7). Four genes were significantly more highly expressed in the flat-leaved Guide accession than in both rod-leaved accessions: the YABBY lamina gene *ZxYAB2*, which fell from 72 normalised counts in Guide to fewer than six in either rod-leaved accession, the auxin/polarity factors *ZxARF3* (ETTIN) and *ZxARF18* and the wall-loosening expansin *ZxEXPA1*, which fell from 213 counts to fewer than twenty.

**Figure 4:**
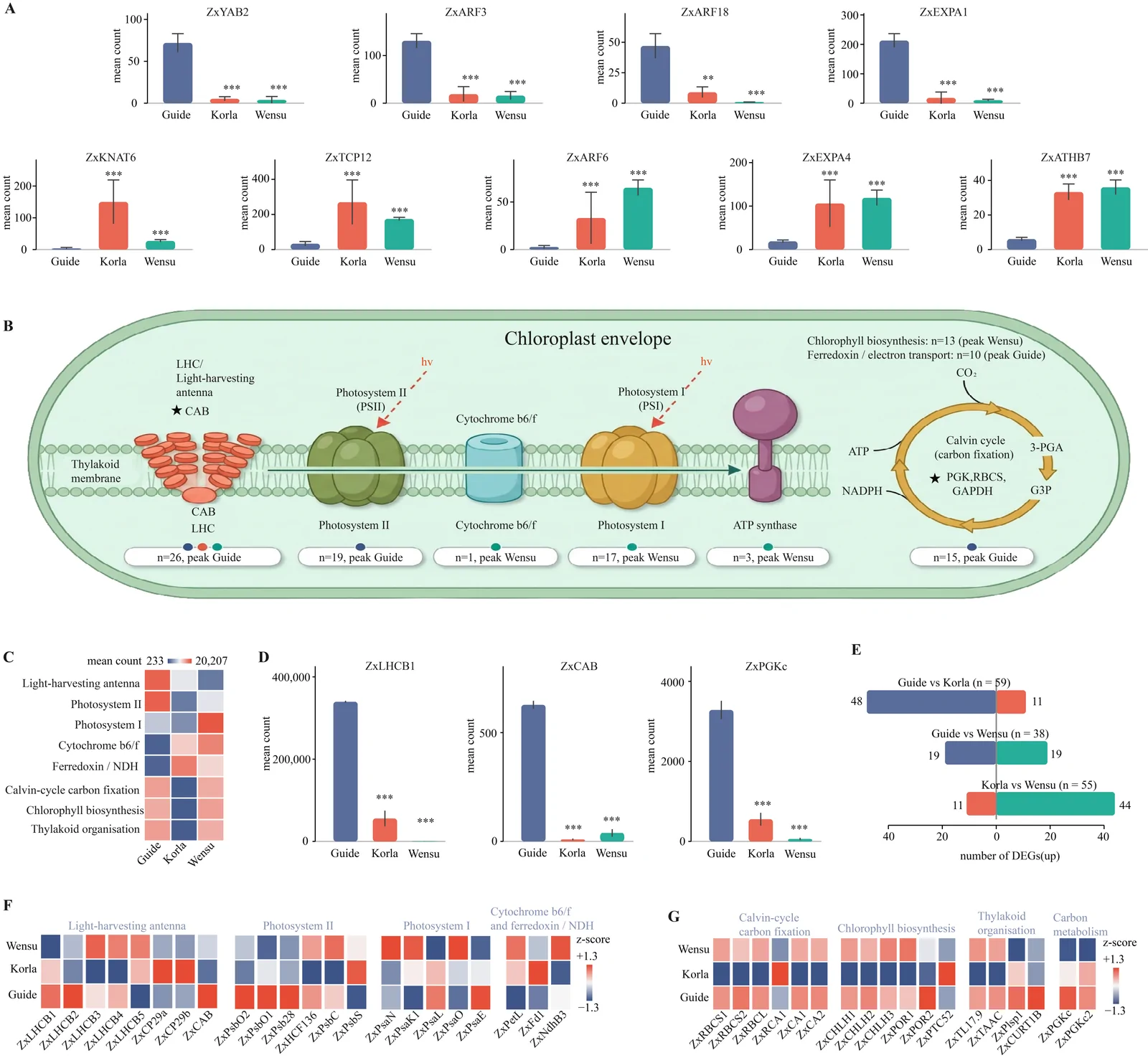
Leaf-development and photosynthesis genes track the flat-versus-rod leaf contrast. (**A**) Expression (mean normalised count ± SD, *n* = 3) of the reciprocal leaf-development module: *ZxYAB2*, *ZxARF3*, *ZxARF18* and *ZxEXPA1* are higher in the flat-leaved Guide accession, whereas *ZxKNAT6*, *ZxTCP12*, *ZxARF6*, *ZxEXPA4* and *ZxATHB7* are higher in both rod-leaved accessions. Asterisks, DESeq2 adjusted *p* versus Guide (*** *p* < 0.001, ** *p* < 0.01 ). (**B**) Schematic of the photosynthetic apparatus with the number of DEGs and the accession of peak expression for each of the eight sub-pathways (102 DEGs in total; only plastid-targeted, photosynthetic transcripts are included). (**C**) Mean expression of each sub-pathway as a row z-score of the log_2_ mean normalised count, with the highest accession mean given at the right. (**D**) Expression of the light-harvesting markers *ZxLHCB1*, *ZxCAB* and the cytosolic phosphoglycerate kinase *ZxPGKc* (mean normalised count ± SD, *n* = 3; *** adjusted *p* < 0.001 versus Guide). (**E**) Numbers of photosynthesis DEGs higher in the first (left) or second (right) accession of each comparison. (**F**,**G**) Row-scaled heat maps of representative photosynthesis transcripts grouped by sub-pathway. Abbreviations are given in Table S7.

Six genes showed the reciprocal, rod-high pattern. The class I KNOX gene *ZxKNAT6* was lowest in the flat-leaved Guide and strongly elevated in both rod-leaved accessions, rising from about four normalised counts to 150 in Korla and 27 in Wensu. The class II TCP factor *ZxTCP12*, which in model species restrains blade proliferation, was likewise rod-high, as were *ZxARF6*, the expansin *ZxEXPA4*, the HD-ZIP I factor *ZxATHB7* and the organ-boundary gene *ZxLBD38*. All ten genes change concordantly in both flat-versus-rod comparisons, and nine of them meet the formal criteria of the leaf-form–associated set: eight are leaf-form–specific, and *ZxKNAT6* is leaf-form–graded, rod-high in both comparisons while reaching its maximum in Korla. *ZxYAB2* shows the same concordant flat-high pattern and is treated with them throughout.

Not every canonical polarity gene tracked leaf form, and the exceptions map cleanly onto the site axis. The class III HD-ZIP adaxial-polarity factor *ZxATHB15* was high in both Guide and the rod-leaved Wensu and reduced only in Korla, which places it in the Korla-specific set rather than the leaf-form set; several BEL1-like and other HD-ZIP genes behaved similarly (Figure 3G; Table S7). The flat-versus-rod difference in these accessions is therefore not accompanied by a simple loss of class III HD-ZIP expression; it is instead associated with a specific YABBY–ARF–expansin module opposed by KNOX, TCP and LBD activity.

### 2.7. Photosynthesis Gene Expression Is Redistributed Among Sub-Pathways, with Antenna Transcripts, CAB and PGK Highest in the Flat-Leaved Accession

One hundred and two DEGs encoded plastid-targeted components of the photosynthetic apparatus, distributed across eight sub-pathways (Figure 4B,C; Table S5). The light-harvesting antenna stood out, its mean expression in Guide exceeding that in either rod-leaved accession by roughly an order of magnitude. Photosystem II, Calvin-cycle carbon fixation and thylakoid organisation also peaked in Guide, whereas photosystem I, cytochrome *b*^6^*f* and chlorophyll biosynthesis peaked in Wensu and ferredoxin/NDH in Korla. Four of the eight sub-pathways therefore peaked in Guide, three in Wensu and one in Korla, so the divergence is a redistribution among components of the apparatus rather than a uniform up- or down-regulation. The redistribution is directional with respect to the harshest site: relative to Korla, photosynthesis DEGs were strongly biased upwards in both Guide and Wensu, whereas Guide and Wensu were balanced against each other (Figure 4E). Carbon fixation is thus better described as Korla-low than as flat-leaf-high, while the antenna signal is specific to the flat-leaved accession.

Two transcripts carried the flat-leaf signal most strongly (Figure 4D; Tables S6 and S7). The chlorophyll *a*-*b* binding protein *ZxCAB* was expressed at 628 normalised counts in Guide against 9 in Korla and 39 in Wensu, and the cytosolic phosphoglycerate kinase *ZxPGKc* at 3286 against 556 and 62. The most abundant antenna transcript of all, *ZxLHCB1*, followed the same direction at a far higher level. All three belong to the leaf-form–associated set: each is significantly higher in the flat-leaved accession than in either rod-leaved accession taken on its own, so that Guide sits at the extreme of the series, and each is leaf-form–graded, the two rod-leaved accessions differing in how far below Guide they lie.

Because a highly expressed, repetitive gene family can be split across several clusters in a de novo assembly, we also examined the two markers at the level of their whole families. Summed across all 39 quantified members, total chlorophyll *a*-*b* binding protein abundance was 4.8-fold higher in Guide than in Korla and 16.7-fold higher than in Wensu, and all three quantified cytosolic phosphoglycerate kinase clusters were likewise highest in Guide. The flat-leaf bias of *CAB* and *PGK* is therefore a property of the gene families as a whole and not of a single assembled cluster. Heat maps of representative transcripts from every sub-pathway (Figure 4F,G) confirm that antenna, photosystem II and carbon-fixation transcripts are highest in Guide as a group, whereas several photosystem I, cytochrome *b*^6^*f* and chlorophyll-biosynthesis transcripts are highest in Wensu.

### 2.8. qPCR Confirms the Expression Estimates and Illustrates the Site Axis

The quantitative reliability of the expression estimates was checked by qPCR on four DEGs chosen to span a wide range of fold changes and assayed on cDNA prepared from the same total RNA as the sequencing libraries, so that the comparison is a technical confirmation of the quantification rather than an independent biological validation (Figure 5A,B; Table S6). Across the twelve gene × comparison points, the two platforms were closely correlated and agreed in direction at every point. As expected, RNA-seq reported the larger absolute fold changes, reflecting its wider dynamic range. Agreement across roughly sixteen log_2_ units indicates that the quantification pipeline reports expression differences accurately over the full range encountered in this study, including the very large differences reported above for *ZxCAB*, *ZxLHCB1* and *ZxPGKc*. *Actin*, the reference gene used for normalisation, was itself stable across the three accessions in the transcriptome data (Table S9).

**Figure 5:**
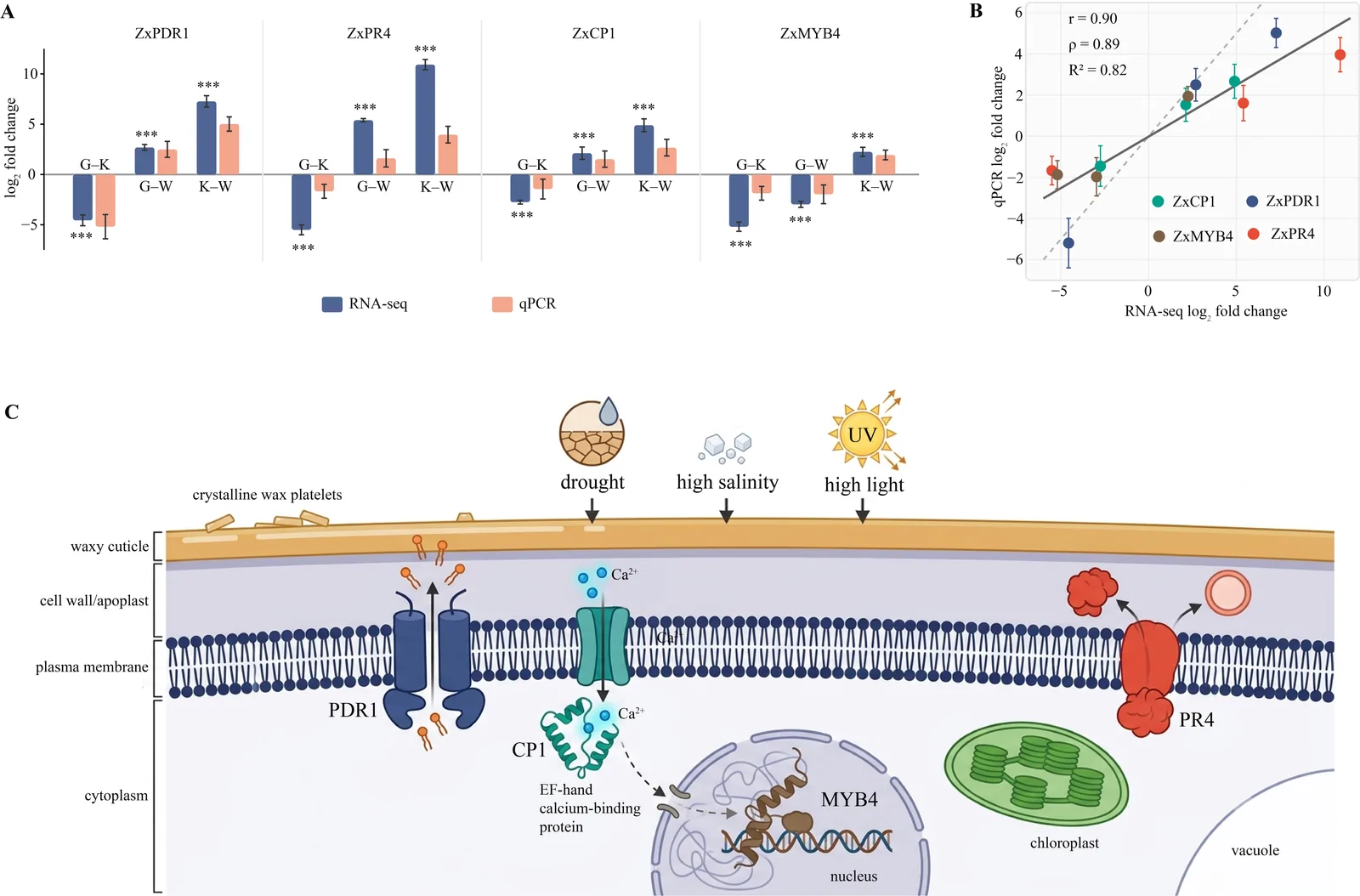
Quantitative RT-PCR confirmation of the expression estimates and the site-associated stress genes. (**A**) RNA-seq and qPCR log_2_ fold changes for the four genes assayed by qPCR in the three pairwise comparisons; direction is relative to the first accession of each pair, and qPCR 2^−ΔΔCt^ values were converted to log_2_ fold changes for direct comparison. The two methods agree in direction at all twelve points. Error bars are, for RNA-seq, the standard error of the difference of the replicate log_2_ means and, for qPCR, the standard error of the log ratio propagated from the SDs of the calibrator and test samples (*n* = 3 per accession). Asterisks above each pair give the DESeq2 adjusted *p* for that comparison (*** *p* < 0.001). (**B**) Correlation between the RNA-seq and qPCR log_2_ fold changes across the twelve points (Pearson *r* = 0.90, *p* < 0.001; Spearman *ρ* = 0.89, *p* < 0.001; *R*^2^ = 0.82); solid line, linear regression; dashed line, *y* = *x*. The correlation shows that the sequencing-based estimates are quantitatively reliable over the full range of fold changes encountered here, the larger absolute values returned by RNA-seq reflecting its wider dynamic range. Normalised counts for the four genes are given at the right; all four peak in the Korla accession. (**C**) Proposed cellular roles of the four genes under desert stress: ZxPDR1 exports wax and cutin precursors to the cuticle, ZxCP1 binds the stress-induced Ca^2+^ signal, ZxMYB4 acts in the nucleus to activate stress and secondary-metabolism genes, and ZxPR4 acts in defence. The scheme is a hypothesis derived from the annotation and expression of the four genes; the interactions shown were not tested here.

The four genes assayed by qPCR are themselves informative, because all peak in the accession from the harshest site: three are Korla-extreme, with Guide intermediate between the two rod-leaved accessions, and the fourth is higher in both rod-leaved accessions but maximal in Korla (Table S6). Their annotations place them squarely in the abiotic-stress arm of the response (Figure 5C): the ABCG/PDR transporter exports wax and cutin precursors to the cuticle, CP1 binds the Ca^2+^ signal generated by ionic and osmotic stress, MYB4 is a transcriptional regulator of stress-responsive and secondary-metabolism genes and PR-4 is a secreted, chitinase-like defence protein. Their coordinated up-regulation in the lowest, brightest and most continental accession, under common-garden conditions, ties the site axis of the transcriptome to the drought, salinity and high-light stress that characterise that source site.

## 3. Discussion

Because the three accessions were raised together from seed in a common garden, the differences recorded here are properties the plants carry with them rather than responses to the environments they came from. Those differences do not form a single gradient. They resolve into two largely independent axes: one that tracks leaf form, separating the flat-leafleted Guide accession from the two rod-leafleted ones and confined to the leaflet blade rather than the petiole, and one that tracks the site of origin and is driven almost entirely by the distinctiveness of the Korla accession. Neither axis is visible in isolation, since any pairwise comparison moves both at once.

The genes on the leaf-form axis correspond closely, in annotation and in the direction of their expression difference, to the leaf-flattening pathway established in model species [14,25]. A flat, dorsiventral leaf requires adaxial–abaxial polarity, lamina outgrowth and sustained proliferation, and the flat-leaved accession was up-regulated for all three processes—the lamina-promoting YABBY gene *ZxYAB2*, the auxin/polarity factors *ZxARF3* (ETTIN) and *ZxARF18* and the wall-loosening expansin *ZxEXPA1* [17,20,22]. Running counter to them, and higher in both rod-leaved accessions, were *ZxKNAT6*, *ZxTCP12*, *ZxLBD38*, *ZxARF6*, *ZxEXPA4* and *ZxATHB7* [25,26]. Each direction is the expected one. KNOX activity must be excluded from the primordium before a determinate, flattened leaf can form [18]; class II TCP factors restrain blade proliferation [21]; LBD genes act at organ boundaries [27]. *ZxKNAT6* warrants a caveat, since in *Arabidopsis*, KNAT6 functions mainly at organ boundaries rather than in meristem maintenance proper, and we therefore read it as an indicator of KNOX-pathway activity rather than as the determinant of indeterminacy [28].

One canonical gene does not follow leaf form, and it is the more informative for that. *ZxATHB15*, a class III HD-ZIP, was high in Guide and equally high in the rod-leaved Wensu, falling only in Korla; it therefore tracks the site rather than the leaf. Pooled with the rest, it would have appeared to contradict the polarity model. Resolved onto the site axis, it indicates instead that the difference between these flat and rod leaves is not a straightforward loss of class III HD-ZIP polarity [15,16] but is associated with the YABBY–ARF–expansin module described above [29,30]. That the module behaves coherently across accessions grown side by side from seed is reason enough to place its members on a list for functional work [8].

The same axis carries a larger signal in photosynthesis: expression of the apparatus as a whole is highest in the flat-leaved accession and lowest in the most extreme one, but the sub-pathways do not scale up and down together [31]. Carbon fixation is better described as Korla-low than as flat-leaf-high, since Guide and Wensu sit at comparable levels, and several sub-pathways peak in Wensu instead. Once these are set aside, the light-harvesting antenna is what remains on the leaf-form axis.

Three features make that antenna signal interpretable. It is very large, *ZxCAB* and *ZxPGKc* standing one to two orders of magnitude above both rod-leaved accessions; it holds at the level of the entire chlorophyll *a*-*b* binding protein family, which rules out a single aberrant contig or an artefact of assembly; and it is independent of the light the plants were grown in. This last point carries the weight of the argument. Elevated *CAB* expression is the classic signature of a shade-acclimated leaf [32,33], and the flat-leaved accession does originate from a shaded valley; had the plants been sampled in the field, the result would have been uninterpretable. Grown under a single light regime, it instead reflects a constitutive difference in how much light-harvesting machinery each accession builds [34,35]. A wider, flatter blade and a greater investment in the machinery to fill it belong together in a leaf whose function is to intercept light rather than to shed it. The second marker converges on the same conclusion from an unrelated direction: cytosolic phosphoglycerate kinase belongs not to the Calvin cycle but to the cytosolic arm of carbon metabolism, acting downstream on the triose phosphates exported from the chloroplast [36]. Its family-wide bias towards the flat leaf therefore adds to the antenna signal rather than restating it, and together the two provide a compact expression signature that can be tested in further individuals and populations [37].

The site axis is of a different character, and one accession accounts for most of it. Korla emerges as the outlier however the data are partitioned—in the correlation structure, the largest DEG comparison, the largest co-expression cluster and the qPCR panel—and Korla is also the site with the most severe climate. The 1121 genes that distinguish it are enriched for stress and defence, transport and transcription-factor and secondary-metabolism functions, and the cuticle, water-relations and drought genes move in the same direction [38]. The four genes assayed by qPCR illustrate the programme concretely: an ABCG/PDR transporter exporting wax and cutin precursors [39,40], an MYB factor acting upstream of stress and secondary-metabolism genes [41,42], a Ca^2+^-binding protein responsive to ionic stress [43] and a secreted PR-4 defence protein. None of the stresses that would account for this programme were present in the common garden, yet the programme was expressed nonetheless, so it is constitutive in these accessions and is maintained in the absence of the stresses that would elicit it [10,11].

Considered together, the two axes describe not a list of independent traits but two coordinated strategies. The accession from the sheltered, shaded, thermally moderate site combines a flat, broad blade with a high investment in light harvesting and with larger, less dense seeds; the two from low, exposed, strongly continental sites combine rod-shaped blades with a transcriptome weighted towards stress and transport and with smaller, denser seeds. The seed contrast is slight—thousand-grain weight barely differs, and only the packing changes—but it runs with the leaf and transcriptome contrasts rather than against them. These data cannot establish whether the traits are genetically coupled or have simply been selected together. What the common-garden design does establish is that the whole combination, and not any single trait within it, differs between populations, and a combination that differs between populations is one on which selection can act [12,13].

Two points frame these results. The leaf-form axis is defined by a single flat-leaved accession set against two rod-leaved ones and is therefore an expression pattern associated with the flat-versus-rod contrast rather than a set of genes shown to determine leaf form in *Z. xanthoxylum*; that the genes recovered are those the leaf-flattening pathway of model species would predict and that they change in the predicted directions provide biological support for the association. The members of the module are accordingly candidate genes associated with the flat-leaved phenotype. The common-garden design likewise establishes that the morphological and transcriptional differences are maintained when the plants are grown away from their source environments, which excludes a direct plastic response to current conditions, although it does not by itself establish them as genetic adaptations to those environments. Both points are most directly addressed by extending the comparison to additional independent flat- and rod-leaved accessions, for which the module reported here provides a compact set of candidates.

## 4. Materials and Methods

### 4.1. Plant Material and Source Habitats

Seeds of three accessions of *Zygophyllum xanthoxylum*, each from a distinct desert habitat, were obtained from the Germplasm Bank of Wild Species, Kunming Institute of Botany, Chinese Academy of Sciences. Accessions are referred to throughout by readable site names, with their permanent collection numbers given in parentheses and in Table 1: Guide, from a hot, dry valley near Guide, Qinghai Province (about 2660 m), where the plants grow beneath a plantation canopy on soft, nutrient-rich soil with adequate water and comparatively low light; Korla, from open gravel (gobi) desert near Korla, Xinjiang (about 946 m), on poor sandy soil under severe drought and full sun; and Wensu, from a sandy roadbed near Wensu, Xinjiang (about 1492 m), also arid and fully sun-exposed. Guide is flat- and broad-leaved, whereas Korla and Wensu are terete- (rod-) leaved.

The three sites differ mainly in altitude, continentality and light. Long-term monthly climate for each site was extracted from the WorldClim version 2 global climate surfaces (about 1 km resolution) using the collection coordinates [44]; the annual mean temperature and the mean temperatures of the warmest and coldest months were used to describe the thermal regime (Table 1) and were cross-checked against the records of the nearest meteorological stations. The two low-altitude Xinjiang sites experience a much wider annual temperature range than the higher-altitude Qinghai site (annual range ΔT ≈ 37.0 °C for Korla and ≈32.5 °C for Wensu, versus ≈24.4 °C for Guide). Soil and light conditions are those recorded at collection.

### 4.2. Seed Morphology

Mature seeds were cleaned and photographed, and the long and short axes of 100 randomly selected seeds per accession were measured individually. Thousand-grain weight was determined by direct weighing. Three derived traits were computed geometrically from the axis lengths (L, long axis; S, short axis): projected area (π/4 · L · S), volume (π/6 · L · S^2^, a prolate-spheroid approximation) and density (single-seed mass divided by the estimated volume). Projected area and volume were also computed for each seed from its own axis lengths, and differences among accessions in the two measured axes and in these per-seed values were tested by one-way analysis of variance followed by Dunnett’s test against Guide (*n* = 100 seeds per accession); the values plotted in Figure 1E are those obtained from the mean axis lengths. Density is computed from the mass of the seed lot and is therefore a single value per accession, and the aspect ratio is a ratio of the same two measurements, so neither was tested separately. The three derived traits are reported as geometric descriptors of seed shape and packing.

### 4.3. Common-Garden Growth and Seedling Morphometrics

Seeds were surface-sterilised in a laminar-flow cabinet by immersion in ≥95% ethanol for 40 s followed by 70–75% ethanol for 5 min, with continuous inversion, and were then rinsed repeatedly in sterile water. At least twenty sterilised seeds per accession were germinated on 1% agar in a growth cabinet; germination required about seven days. Seedlings of similar vigour were transplanted into pots containing vermiculite and nutrient soil (1:1, *v*/*v*) and grown together under identical light, temperature and watering regimes. At least ten plants of each accession were raised in this way. Seedlings were photographed 7 days and 30 days after transplanting, and adaxial and abaxial views of representative leaves were recorded. One month after transplanting, ten independent plants of each accession were sampled, one morphologically uniform, fully expanded leaf from the middle of the shoot being taken from each plant, and the length, width and width-to-length ratio of the leaflet blade and of the petiole were measured on it. Each plant therefore contributed a single value per trait, and the plant and not the leaf was treated as the statistical unit (*n* = 10 plants per accession). Three of these ten plants per accession were then selected at random to provide the three biological replicates used for RNA extraction and transcriptome sequencing (Section 4.4), the leaf material for sequencing being taken at the same developmental stage and from the same position on the shoot. Each leaf of *Z. xanthoxylum* bears two leaflets; the blade measured here is therefore the leaflet, and “leaf” and “leaflet” are used accordingly below.

### 4.4. RNA Extraction, Library Construction and Sequencing

Total RNA was extracted from leaf tissue of each of the nine samples (three accessions × three biological replicates) harvested one month after transplanting. RNA integrity and purity were assessed by agarose-gel electrophoresis, NanoDrop spectrophotometry (Thermo Fisher Scientific, Wilmington, DE, USA), Qubit fluorometry (Thermo Fisher Scientific, Waltham, MA, USA) and an Agilent 2100 Bioanalyzer (Agilent Technologies, Santa Clara, CA, USA). Poly(A)^+^ RNA was enriched with oligo(dT) magnetic beads, fragmented and used to construct non-strand-specific paired-end libraries; libraries were quantified with a Qubit 2.0 fluorometer (Thermo Fisher Scientific, Waltham, MA, USA), their insert size checked on an Agilent 2100 Bioanalyzer and their effective concentration determined by quantitative PCR before pooling. Sequencing was performed on the Illumina HiSeq 2000 platform (Illumina, Inc., San Diego, CA, USA). Raw reads were quality-filtered with Trimmomatic v0.36 (ILLUMINACLIP:TruSeq3-PE:2:30:10, LEADING:3, TRAILING:3, SLIDINGWINDOW:4:15, MINLEN:50) to remove adapters and low-quality bases, and reads with more than 5% ambiguous bases were discarded [45]; per-library read counts, Q20, Q30, GC content and error rate are given in Table S1.

### 4.5. De Novo Assembly, Annotation and Expression Quantification

Clean reads from all nine libraries were pooled and assembled de novo with Trinity v2.4.0 (--min_contig_length 200, --min_kmer_cov 2, k-mer size 25, with in silico normalisation of read coverage to 50×) [46]. The resulting transcripts were hierarchically clustered with Corset v1.06 (default settings, -m 10 -d 0.3) [47], which groups transcripts that share multi-mapping reads and then separates transcripts with divergent expression profiles; each final cluster was treated as a gene, and the longest transcript in a cluster was retained as its representative (unigene) sequence. Assembly completeness was assessed with BUSCO v5.4.3 in transcriptome mode against the viridiplantae_odb10 single-copy orthologue set (*n* = 425) [48]. Unigenes were annotated by sequence similarity search with DIAMOND v0.9.24 (blastx, E-value threshold 1 × 10^−5^, best hit retained) against the NCBI non-redundant protein (NR) database, Swiss-Prot and KOG, and with BLAST+ v2.9.0 (blastn, E-value threshold 1 × 10^−5^) against the NCBI nucleotide (NT) database; Gene Ontology (GO) terms and KEGG orthology assignments were transferred from the best-scoring annotated hit [49,50]. Expression was quantified by mapping the clean reads of each library back to the unigene set with bowtie2 v2.3.4.1 within RSEM v1.3.0 (default parameters), which reports expected counts and transcripts per million (TPM) [51]. Expected counts were used for differential-expression testing and, after DESeq2 median-of-ratios normalisation, for all expression values reported here; TPM was used for the whole-transcriptome sample-correlation analysis.

### 4.6. Differential Expression, Axis Partitioning and Co-Expression Clustering

Differential expression was tested with DESeq2 v1.22.2 [52] in R v3.5.2 (Wald test on the negative-binomial fit, Benjamini–Hochberg correction) for the three pairwise comparisons Guide versus Korla, Guide versus Wensu and Korla versus Wensu. A gene was called differentially expressed (DEG) when it satisfied three criteria: |log_2_ fold change| ≥ 2, Benjamini–Hochberg adjusted *p* < 0.01 and a non-zero count in every replicate of both accessions of the comparison, the last requirement excluding genes supported only by presence/absence calls. Throughout, fold-change direction is expressed relative to the first accession named in each comparison, so that a positive log_2_ fold change denotes higher expression in that accession.

Because global divergence proved to be dominated by one accession (Section 2.4), the DEGs were partitioned along the axes that the design defines. The leaf-form–associated set comprises genes differentially expressed in the same direction in both Guide versus Korla and Guide versus Wensu, so that the flat-leaved accession lies at one extreme relative to both rod-leaved accessions. Within it, leaf-form–specific genes are additionally not differentially expressed between Korla and Wensu, whereas leaf-form–graded genes also differ in magnitude between the two rod-leaved accessions. The Korla-specific and Wensu-specific sets comprise genes that set one rod-leaved accession apart from the other two, and the Korla-extreme set genes differentially expressed in all three comparisons with Guide intermediate between the two rod-leaved accessions.

For co-expression analysis, the DEGs quantified in all three accessions were summarised as the mean normalised count per accession, log_2_-transformed, converted to a row z-score and grouped by k-means clustering (R stats::kmeans, Hartigan–Wong algorithm, nstart = 50, seed 1). Six clusters were retained on the basis of the within-cluster sum of squares, which declined steeply up to k = 6 and gradually thereafter. Functional categories (transcription factor, protein kinase and signalling, transport, hormone, stress and defence, secondary metabolism, cell wall/cuticle/wax) and transcription-factor families were assigned from the best Swiss-Prot or NR description and are therefore descriptive and may overlap. Over-representation of functional categories and curated gene sets, both within each axis set and among clusters, was tested against the remaining DEGs by two-sided Fisher’s exact test (R stats::fisher.test).

### 4.7. Curated Gene Sets

Three gene sets were curated manually from the Swiss-Prot and NR descriptions of the DEGs. The photosynthesis set was restricted to plastid-targeted components of the photosynthetic apparatus and divided into eight sub-pathways: light-harvesting antenna, photosystem II, photosystem I, cytochrome b_6_f, ferredoxin and NDH, Calvin-cycle carbon fixation, chlorophyll biosynthesis and thylakoid organisation. Transcripts whose best hit was mitochondrial ATP synthase, cytosolic glyceraldehyde-3-phosphate dehydrogenase or fructose-bisphosphate aldolase, cytochrome c_6_, sulfite reductase, ferredoxin-dependent glutamate synthase, HDS/IspG or a non-photosynthetic ferredoxin–NADP reductase isozyme were excluded. The leaf-development set comprised the YABBY, KNOX/BLH, HD-ZIP, ARF, expansin, TCP, GRF and LBD families; the cuticle, water-relations and drought set comprised wax and cutin biosynthesis, GDSL esterases, lipid-transfer proteins, aquaporins, LEA proteins and dehydrins, ABA-related regulators and osmolyte-biosynthesis genes. Gene symbols prefixed “Zx” denote the best database hit and indicate putative homology only; where several clusters matched the same protein they were numbered consecutively. Identifiers, best hits, E-values, clusters and classifications are given in Table S7.

### 4.8. Quantitative RT-PCR Confirmation

Four DEGs spanning a wide range of fold changes were assayed by quantitative real-time PCR (qPCR): an ABCG/PDR-type transporter (ZxPDR1, Cluster-29589.56456), a pathogenesis-related protein PR-4 (ZxPR4, Cluster-29589.31587), a Ca^2+^-binding protein CP1 (ZxCP1, Cluster-29589.43057) and an MYB4-type transcription factor (ZxMYB4, Cluster-23309.2). Gene-specific primers were designed against the assembled unigene sequences and are listed in Table S10. First-strand cDNA was synthesised from the same total RNA used for library construction with the PrimeScript RT Reagent Kit with gDNA Eraser (TaKaRa, Dalian, China), and qPCR was performed with TB Green Premix Ex Taq II (Takara Bio Inc., Kusatsu, Shiga, Japan) on a LightCycler 480 II system (Roche Diagnostics GmbH, Mannheim, Germany), in both cases following the manufacturer’s instructions. Product specificity was confirmed by melting-curve analysis. Each of the three biological replicates per accession was measured in three technical replicates, which were averaged before analysis. Expression was normalised to actin, which was chosen as the reference gene because it is one of the most widely used internal controls in plant qPCR and because its stability across the three accessions could be verified directly in the transcriptome data: the most abundant near-full-length actin transcript of the assembly (Swiss-Prot hit Actin-7) varied only 1.24-fold between the highest and the lowest accession mean, with a coefficient of variation of 10.8% among the three accession means against 1.5–4.2% among replicates within an accession (Table S9). Expression was calculated by the 2^−ΔΔCt^ method [53], with the first accession of each pairwise comparison as the calibrator. Relative-expression values were converted to log_2_ fold changes in the same pairwise directions as the RNA-seq analysis. Differences between accessions were tested on ΔCt values by one-way analysis of variance followed by Tukey’s HSD test, and agreement between the two platforms was assessed by Pearson and Spearman correlation and by linear regression.

### 4.9. Statistical Analysis

Morphological traits are reported as means; the seed traits were tested on the per-seed measurements as described in Section 4.2. Differences among accessions in leaflet-blade and petiole dimensions were tested by one-way analysis of variance followed by Dunnett’s test, with the flat-leaved Guide accession as the reference group; asterisks in Figure 2 denote each rod-leaved accession versus Guide. The significance of expression differences derives from the DESeq2 adjusted *p*-values described in Section 4.6. Over-representation of functional categories and curated gene sets was tested by Fisher’s exact test. All downstream analyses and figures were produced with custom scripts in R v3.5.2 (DESeq2 v1.22.2, ggplot2 v3.3.5) and Python v3.9.7 (numpy v1.21.2, pandas v1.3.3, scipy v1.7.1, matplotlib v3.5.1).

## 5. Conclusions

Three habitat-divergent accessions of *Zygophyllum xanthoxylum*, raised together from seed, differ constitutively in leaflet-blade shape—flat and broad in an accession from a shaded, high-altitude valley, narrow and rod-like in two from arid, full-sun sites—while their petioles are conserved. De novo transcriptome comparison shows that this contrast is accompanied by coordinated expression differences. A reciprocal leaf-development module, in which the lamina-associated genes *YAB2*, *ARF3*, *ARF18* and *EXPA1* are opposed by the rod-associated genes *KNAT6*, *TCP12*, *ARF6*, *EXPA4*, *ATHB7* and *LBD38*, tracks the flat-versus-rod contrast, and photosynthesis gene expression is significantly concentrated at the flat-leaved end of the series and redistributed among sub-pathways, with light-harvesting transcript abundance 4.8- to 16.7-fold higher in the flat-leaved accession at the level of the whole gene family. A second, site-associated axis sets the accession from the harshest habitat apart and is enriched for stress, transport, regulatory and secondary-metabolism genes, including four genes confirmed by qPCR; separating this axis from the leaf-form axis is necessary to read the transcriptome correctly. These results provide a first molecular portrait of flat- versus rod-shaped leaflet differentiation in *Z. xanthoxylum*, and they identify *CAB* and *PGK*, together with *YAB2*, *KNAT6* and the associated leaf-development genes, as candidate genes associated with the flat-leaved phenotype, to be tested in additional independent flat- and rod-leaved accessions and by functional study.

## Figures and Tables

**Figure 1:**
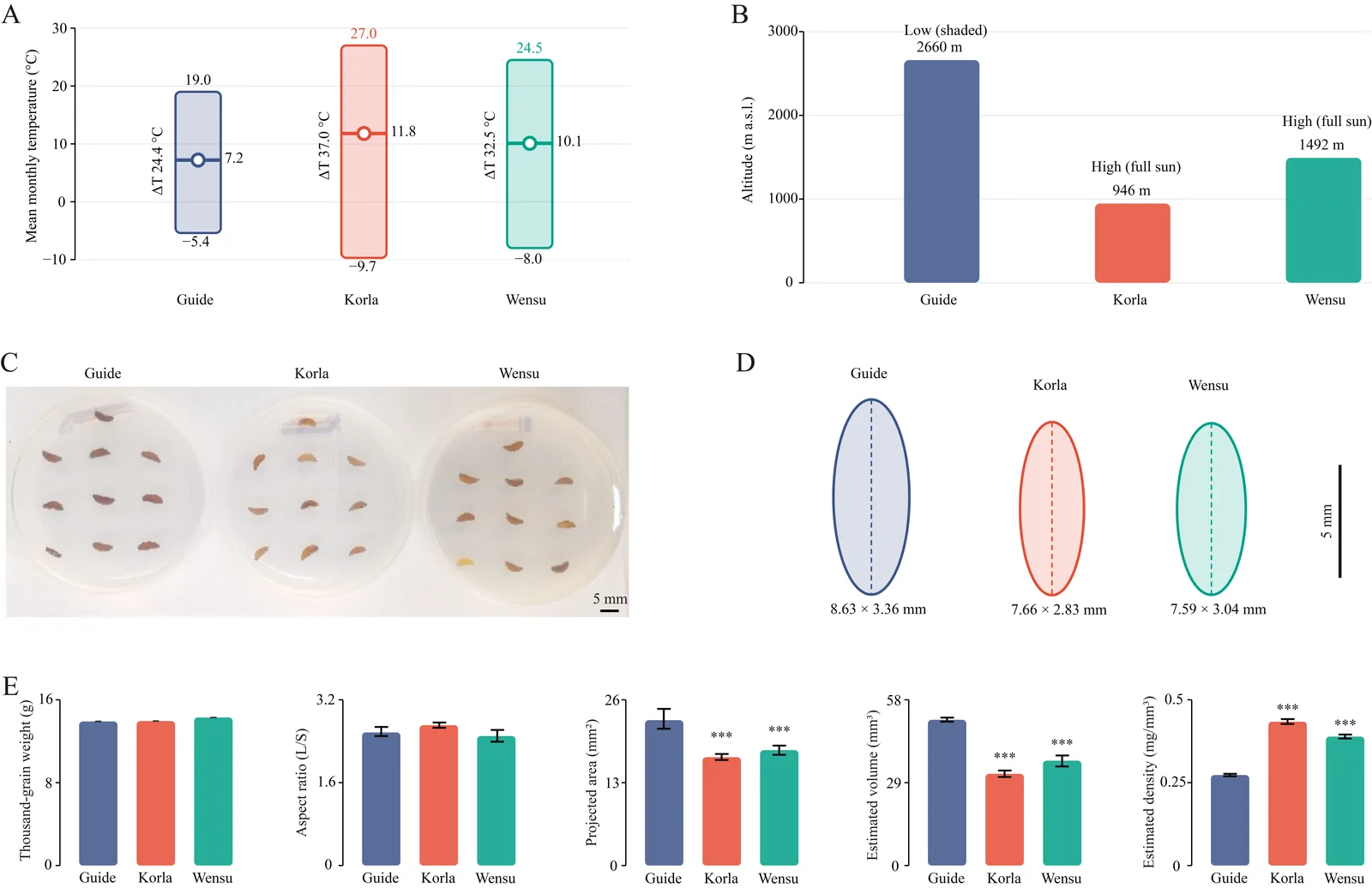
Source-habitat environment and seed morphology of three habitat-divergent accessions of *Zygophyllum xanthoxylum*. Accessions and colour code throughout Figure 1, Figure 2, Figure 3, Figure 4 and Figure 5: Guide (Chensl1200; Guide, Qinghai; flat, broad leaflets) navy; Korla (LiZJ1402; Korla, Xinjiang; terete leaflets) red; Wensu (LiZJ0039; Wensu, Xinjiang; terete leaflets) teal. Collection numbers and site details are given in Table 1. (**A**) Temperature regime of the three sites: each bar spans the mean temperature of the coldest to the warmest month, the horizontal line and open circle mark the annual mean and ΔT is the annual range. Values in °C were obtained for each site’s coordinates from the WorldClim v2 climate surfaces and cross-checked against nearby meteorological-station records. (**B**) Altitude and the qualitative light environment recorded at collection. (**C**) Representative cleaned seeds of each accession. (**D**) Mean seed outlines drawn to scale from the measured long × short axes; dashed line, long axis; scale bar, 5 mm. (**E**) Seed size, shape and packing: thousand-grain weight (mean ± SD), aspect ratio, projected area, estimated volume and estimated density. Thousand-grain weight is nearly identical across accessions, but estimated volume differs 1.6-fold, so Guide has the largest, least dense seeds and Korla the densest. Projected area, volume and density are geometric estimates derived from the mean axis lengths and the thousand-grain weight. Every panel carries a y-axis with tick marks. Error bars on the thousand-grain weight are the SD of three independent weighings of the seed lot; the other four panels plot the values obtained from the mean axis lengths and therefore carry no error bars. Asterisks denote Dunnett’s test against Guide (*** *p* < 0.001); the seed axes and the derived projected area and volume were tested on the per-seed measurements (*n* = 100).

**Table 1:** The three accessions of *Zygophyllum xanthoxylum* and their source habitats.

Accession	Locality	Altitude (m)	Warmest/Coldest Month; ΔT (°C)	Habitat and Light	Leaflet Form
Guide	Guide, Qinghai	2660	19.0/−5.4; 24.4	Hot, dry valley beneath a plantation canopy; soft, nutrient-rich soil, adequate water; low light	Flat, broad
Korla	Korla, Xinjiang	946	27.0/−9.7; 37.0	Open gravel (gobi) desert; poor sandy soil, severe drought; full sun	Terete (rod)
Wensu	Wensu, Xinjiang	1492	24.5/−8.0; 32.5	Open sandy roadbed; arid; full sun	Terete (rod)

Climate values were extracted from the WorldClim v2 surfaces for each site’s coordinates and cross-checked against nearby meteorological stations. ΔT, difference between the mean temperatures of the warmest and coldest months. Habitat, soil and light descriptions are those recorded at collection.

## Data Availability

The raw sequence reads generated in this study have been deposited in the NCBI Sequence Read Archive under BioProject accession PRJNA1511602 (https://submit.ncbi.nlm.nih.gov/subs/sra/SUB16402878/overview) (accessed on 21 September 2026).

## Supplementary Materials

The following supporting information can be downloaded at https://www.mdpi.com/article/10.3390/ijms27198537/s1.
