# Supplementary material for: Common-Garden Transcriptomes Distinguish Leaf-Form-Associated from Site-Associated Expression in Zygophyllum xanthoxylum
Source: Int J Mol Sci. 2026 Sep 24;27(19):8537. doi: 10.3390/ijms27198537 (PMC13665354; doi:10.3390/ijms27198537)
Supplement: Supplementary file 1 [file ijms-27-08537-s001.zip › ijms-4530696-supplementary.pdf]

Supplementary Information

Common-garden transcriptomes distinguish leaf-form-associated from site-associated expression in Zygophyllum xanthoxylum

Xiaoyang Wang [1,2], Wenjing Shen [3], Xingshun Wang [3], Duanyang Yuan [3], Qin Lu [4], Zhentao Ren [5] and Ming Zhu [3,*]

[1] Biological and Medical Metrology Laboratory, Beijing Institute of Metrology, Beijing 100029, China

[2] College of Life and Environmental Sciences, Minzu University of China, Beijing 100081, China

[3] Yunnan Open University, Kunming 650223, China

[4] Institute of Highland Forest Science, Chinese Academy of Forestry, Kunming 650224, China; lqin@caf.ac.cn [5]

Institute of Food and Nutrition Development, Ministry of Agriculture and Rural Affairs, Beijing 100081, China

[*]Corresponding author: lyanzhu0@126.com

Supplementary Tables

Table S1. Sequencing output and quality for the nine leaf libraries.

Accession	Raw reads	Clean reads	Q20 (%)	Q30 (%)	GC (%)	Error (%)
Guide	51,069,736	50,135,808	100	99.57	46.0	0.02
Guide	51,857,864	50,824,592	100	99.58	45.5	0.02
Guide	51,928,764	50,868,508	100	99.56	45.5	0.02
Korla	42,091,779	32,694,150	100	99.58	45.0	0.02
Korla	49,108,922	48,143,874	100	99.57	45.0	0.02
Korla	51,594,792	50,484,242	100	99.51	45.0	0.02
Wensu	51,094,820	50,116,274	100	99.54	46.0	0.02
Wensu	51,260,648	50,300,588	100	99.59	45.5	0.02
Wensu	50,776,868	49,848,530	100	99.57	46.5	0.02

All libraries reached Q20 = 100 % and Q30 > 99.5 % with an error rate of 0.02 %. Library LiZJ1402_1 retained a smaller proportion of clean reads after filtering but met every quality criterion and was retained.

Table S2. Assembly and functional-annotation statistics for the *Zygophyllum xanthoxylum* leaf transcriptome.

Statistic	Value
Genes (Corset clusters)	118,214
Total assembled length	163.7 Mb

Statistic	Value
Mean gene length	1,385 bp
Median gene length	1,056 bp
N50	1,993 bp
Longest gene	14,792 bp
Genes ≥ 500 bp	92,502 (78.2 %)
Genes ≥ 1 kb	61,516 (52.0 %)
BUSCO complete (viridiplantae_odb10, n = 425)	423 (99.5 %)
of which single-copy	102 (24.0 %)
of which duplicated	321 (75.5 %)
BUSCO fragmented / missing	1 (0.2 %) / 1 (0.2 %)
Annotated in NR	90,670 (76.7 %)
Annotated in NT	68,461 (57.9 %)
Annotated in Swiss-Prot	75,183 (63.6 %)
Annotated in KOG	56,760 (48.0 %)
Annotated in at least one database	93,215 (78.9 %)

The high proportion of duplicated BUSCOs is expected for a clustered de novo transcriptome, in which allelic and splice variants of the same locus may be retained as separate clusters.

Table S3. Gene Ontology terms significantly enriched ($P < 0.01$) in each comparison.

Comparison	Biological process	Molecular function	Cellular component
Guide vs Korla	273	85	85
Guide vs Wensu	84	48	28
Korla vs Wensu	269	95	36

Terms describing responses to chemical, endogenous, hormonal and external stimuli, and to oxygen-containing compounds, were among the most significant biological-process terms in all three comparisons. In Guide versus Korla the most significant molecular-function terms were quercetin 3-O-glucosyltransferase activity (GO:0080043), UDP-glucosyltransferase activity (GO:0035251) and dioxygenase activity (GO:0051213), and the most significant cellular-component terms were cell periphery (GO:0071944), plastid stroma (GO:0009532) and plant-type cell wall (GO:0009505). Representative terms are plotted in Supplementary Figure S5.

Table S4. KEGG pathways significantly enriched ($P < 0.01$) in each comparison.

Comparison	n	Enriched pathways
Guide vs Korla	10	Biosynthesis of secondary metabolites; Metabolic pathways; Carbon metabolism; Linoleic acid metabolism; Terpenoid backbone biosynthesis; α-Linolenic acid metabolism; Glycine, serine and threonine metabolism; Taurine and hypotaurine metabolism; Carbon fixation in photosynthetic organisms; Synthesis and degradation of ketone bodies
Guide vs Wensu	8	Biosynthesis of secondary metabolites; Metabolic pathways; Glyoxylate and dicarboxylate metabolism; Zeatin biosynthesis; Peroxisome; Photosynthesis – antenna proteins; Lipoic acid metabolism; Carotenoid biosynthesis
Korla vs Wensu	17	Biosynthesis of secondary metabolites; Metabolic pathways; Flavonoid biosynthesis; Pyruvate metabolism; Ascorbate and aldarate metabolism; Linoleic acid metabolism; Stilbenoid, diarylheptanoid and gingerol biosynthesis; β-Alanine metabolism; α-Linolenic acid metabolism; Alanine, aspartate and glutamate metabolism; Nitrogen metabolism; Amino sugar and nucleotide sugar metabolism; Lysine degradation; Porphyrin metabolism; Carbon metabolism; Arginine and proline metabolism; Taurine and hypotaurine metabolism

Biosynthesis of secondary metabolites and metabolic pathways were the two most significant pathways in every comparison. Photosynthesis-related pathways (in bold) were enriched only in the comparisons involving the flat-leaved Guide accession. The full presence/absence matrix is shown in Supplementary Figure S6.

Table S5. Photosynthesis-associated DEGs by sub-pathway.

Sub-pathway	DEGs	Quantified	Guide	Korla	Wensu	Peak
Light-harvesting antenna	26	18	20,207	3,491	1,009	Guide
Photosystem II	19	10	647	95	204	Guide
Photosystem I	17	10	1,227	890	3,825	Wensu
Cytochrome b_6f	1	1	0	87	452	Wensu
Ferredoxin / NDH	3	2	33	233	126	Korla
Calvin-cycle carbon fixation	15	8	16,272	3,238	14,959	Guide
Chlorophyll biosynthesis	12	9	2,140	520	2,285	Wensu
Thylakoid organisation	9	6	235	58	214	Guide
Total	102	64				

"DEGs" is the number of photosynthesis-associated genes differentially expressed in at least one comparison; "Quantified" is the subset for which normalised expression estimates were available for all three accessions, on which the mean normalised counts for Guide, Korla and Wensu are based; a mean of 0 denotes a transcript that was not detected in any library of that accession, as is the case for the single cytochrome b6f transcript in Guide. Only plastid-targeted, genuinely photosynthetic transcripts are included; transcripts whose best hit was mitochondrial ATP synthase, cytosolic glyceraldehyde-3-phosphate dehydrogenase or fructose-bisphosphate aldolase, cytochrome c_6, sulfite reductase, ferredoxin-dependent glutamate synthase, HDS/IspG or a non-photosynthetic ferredoxin–NADP reductase isozyme were excluded.

Table S5 (continued). Numbers of photosynthesis DEGs higher in each accession of a pair.

Sub-pathway	Guide vs Korla	Guide vs Wensu	Korla vs Wensu
Light-harvesting antenna	9 / 5	5 / 7	4 / 12
Photosystem II	10 / 0	6 / 3	1 / 6
Photosystem I	6 / 1	1 / 5	1 / 9
Cytochrome b_6f	0 / 0	0 / 0	0 / 1
Ferredoxin / NDH	1 / 1	0 / 1	1 / 0
Calvin-cycle carbon fixation	12 / 2	2 / 1	1 / 5
Chlorophyll biosynthesis	5 / 1	3 / 0	2 / 8
Thylakoid organisation	5 / 1	2 / 2	1 / 3
Total	48 / 11	19 / 19	11 / 44

Values are the numbers of DEGs higher in the first / second accession of each pair. Totals match Figure 4E.

Table S6. Core leaf-form markers and the qPCR confirmation set.

Gene	Identifier	Guide	Korla	Wensu	log_2FC G–K (adj. *P*)	log_2FC G–W (adj. *P*)	qPCR log_2FC G–K / G–W / K–W
ZxLHCB1	Cluster-29589.27121	339,558	55,855	666	2.52 (5.8×10^{-47})	8.79 ($< 1 \times 10^{-300}$)	—
ZxCAB	Cluster-29589.27918	628	9	39	5.97 (1.3×10^{-73})	3.81 (1.6×10^{-55})	—
ZxPGKc	Cluster-29589.23198	3,286	556	62	2.46 (5.6×10^{-64})	5.52 (6.6×10^{-54})	—
ZxPDR1	Cluster-29589.56456	82	1,711	11	−4.56 (2.6×10^{-18})	2.69 (2.5×10^{-10})	−5.19 / 2.50 / 5.02
ZxPR4	Cluster-29589.31587	471	19,056	10	−5.51 (1.6×10^{-32})	5.40 (2.8×10^{-78})	−1.67 / 1.61 / 3.96
ZxCP1	Cluster-29589.43057	127	781	26	−2.75 (8.8×10^{-33})	2.12 (1.9×10^{-4})	−1.45 / 1.53 / 2.67
ZxMYB4	Cluster-23309.2	11	348	72	−5.20 (2.6×10^{-19})	−2.97 (1.2×10^{-11})	−1.87 / −1.97 / 1.94

Expression values are mean normalised counts (n = 3). Fold-change direction is relative to Guide for the G–K and G–W comparisons and relative to Korla for K–W. The three markers of the flat-leaved form are given first; the four genes of the qPCR confirmation set, all of which peak in Korla, follow. qPCR values are log2 fold changes converted from 2−ΔΔCt and follow the same direction convention as the sequencing-based values; all twelve agree in direction with RNA-seq.

Table S7. Gene abbreviations, best database hit, co-expression cluster, axis assignment and functional classification for every gene shown in Figures 3–5.

Abbrev.	Best hit description	Gene ID	Accession	E-value	Figure	Cluster	Axis	Classification
ZxARF16	Auxin response factor 16	Cluster-29589.66953	Q93YR9.1	3e-56	3G	C4	Other	Leaf development – ARF
ZxARF18	Auxin response factor 18	Cluster-25730.0	Q9C5W9.1	3e-191	4A, 3G	C2	Leaf-form (specific)	Leaf development – ARF
ZxARF19	Auxin response factor 19	Cluster-29589.16299	Q8RYC8.2	0e+00	3G	C2	Other	Leaf development – ARF
ZxARF3	Auxin response factor 3	Cluster-29589.10282	O23661.2	2e-157	4A, 3G	C1	Leaf-form (specific)	Leaf development – ARF
ZxARF6	Auxin response factor 6	Cluster-29589.9981	Q9ZTX8.2	1e-29	4A, 3G	C5	Leaf-form (specific)	Leaf development – ARF
ZxEXLB1	Expansin-like B1	Cluster-29589.65134	O23547.2	6e-75	3G	C4	Korla-specific	Leaf development – Expansin
ZxEXPA1	Expansin-A1	Cluster-29589.27778	Q9C554.1	4e-67	4A, 3G	C1	Leaf-form (specific)	Leaf development – Expansin
ZxEXPA15a	Expansin-A15	Cluster-30847.0	O80622.2	8e-116	3G	C3	Korla-specific	Leaf development – Expansin
ZxEXPA15b	Expansin-A15	Cluster-29589.29120	O80622.2	1e-108	3G	C4	Other	Leaf development – Expansin
ZxEXPA4	Expansin-A4	Cluster-29589.37905	O48818.1	2e-26	4A, 3G	C6	Leaf-form (specific)	Leaf development – Expansin
ZxGRF7	Growth-regulating factor 7	Cluster-29589.5312	Q9FJB8.1	1e-05	3G	C1	Other	Leaf development – GRF
ZxATHB12	Homeobox-leucine zipper protein ATHB-12	Cluster-29589.45395	Q9M276.1	1e-30	3G	C4	Korla-specific	Leaf development – HD-ZIP
ZxATHB15	Homeobox-leucine zipper protein ATHB-15	Cluster-29589.38526	Q9ZU11.1	0e+00	3G	C3	Korla-specific	Leaf development – HD-ZIP
ZxATHB16	Homeobox-leucine zipper protein ATHB-16	Cluster-29589.36883	Q940J1.2	2e-19	3G	C4	Korla-specific	Leaf development – HD-ZIP
ZxATHB6	Homeobox-leucine zipper protein ATHB-6	Cluster-29589.34115	P46668.1	5e-37	3G	C4	Other	Leaf development – HD-ZIP
ZxATHB7	Homeobox-leucine zipper protein ATHB-7	Cluster-29589.59654	P46897.2	2e-21	4A, 3G	C6	Leaf-form (specific)	Leaf development – HD-ZIP
ZxHAT14	Homeobox-leucine zipper protein HAT14	Cluster-27679.0	P46665.3	2e-55	3G	C4	Korla-specific	Leaf development – HD-ZIP
ZxHAT22	Homeobox-leucine zipper protein HAT22	Cluster-29589.64639	P46604.1	6e-47	3G	C4	Korla-specific	Leaf development – HD-ZIP
ZxBLH1a	BEL1-like homeodomain protein 1	Cluster-29589.30500	Q9SJ56.1	2e-33	3G	C4	Korla-specific	Leaf development – KNOX/BLH
ZxBLH1b	BEL1-like homeodomain protein 1	Cluster-29589.30505	Q9SJ56.1	4e-20	3G	C6	Leaf-form (specific)	Leaf development – KNOX/BLH

Abbrev.	Best hit description	Gene ID	Accession	E-value	Figure	Cluster	Axis	Classification
ZxBLH1c	BEL1-like homeodomain protein 1	Cluster-29589.30528	Q9SJ56.1	4e-43	3G	C4	Korla-specific	Leaf development – KNOX/BLH
ZxBLH3	BEL1-like homeodomain protein 3	Cluster-29589.15124	Q9FWS9.1	1e-87	3G	C3	Korla-specific	Leaf development – KNOX/BLH
ZxKNAT6	Homeobox protein knotted-1-like 6	Cluster-18511.0	Q84JS6.1	2e-47	4A, 3G	C6	Leaf-form (graded)	Leaf development – KNOX/BLH
ZxLBD11	LOB domain-containing protein 11	Cluster-838.1	Q9SK08.2	3e-41	3G	C4	Other	Leaf development – LBD
ZxLBD38	LOB domain-containing protein 38	Cluster-25399.0	Q9SN23.1	2e-46	3G	C6	Leaf-form (specific)	Leaf development – LBD
ZxLBD42	LOB domain-containing protein 42	Cluster-22659.0	Q9CA30.1	2e-50	3G	C5	Wensu-specific	Leaf development – LBD
ZxTCP12	Transcription factor TCP12	Cluster-29589.57899	A0AQW4.1	1e-20	4A, 3G	C6	Leaf-form (specific)	Leaf development – TCP
ZxYAB2	Putative axial regulator YABBY 2	Cluster-29589.41545	Q9XFB0.1	1e-33	4A, 3G	C1	Other	Leaf development – YABBY
ZxABI5L7	ABSCISIC ACID-INSENSITIVE 5-like protein 7	Cluster-29589.30896	Q9M7Q2.1	7e-65	3H	C1	Leaf-form (specific)	Cuticle / water / drought – ABA / drought regulator
ZxCYP707A1	Abscisic acid 8'-hydroxylase CYP707A1	Cluster-29589.61918	A9QNE7.1	7e-202	3H	C5	Leaf-form (specific)	Cuticle / water / drought – ABA / drought regulator
ZxNCED2	9-cis-epoxycarotenoid dioxygenase NCED2, chloroplastic	Cluster-30435.0	K4CJJ1.1	2e-260	3H	C4	Korla-specific	Cuticle / water / drought – ABA / drought regulator
ZxPYL4	Abscisic acid receptor PYL4	Cluster-29589.14805	O80920.1	4e-66	3H	C3	Korla-specific	Cuticle / water / drought – ABA / drought regulator
ZxZEP	Zeaxanthin epoxidase, chloroplastic	Cluster-29589.28370	P93236.1	8e-05	3H	C1	Leaf-form (specific)	Cuticle / water / drought – ABA / drought regulator
ZxNIP3;1	Aquaporin NIP3-1	Cluster-29589.47396	Q0IWF3.2	1e-89	3H	C3	Korla-specific	Cuticle / water / drought – Aquaporin
ZxNIP6;1	Aquaporin NIP6-1	Cluster-20256.0	Q9SAI4.1	1e-90	3H	C4	Korla-specific	Cuticle / water / drought – Aquaporin
ZxPIP2;5	Probable aquaporin PIP2-5	Cluster-29589.26895	Q9SV31.1	1e-128	3H	C6	Leaf-form (specific)	Cuticle / water / drought – Aquaporin
ZxTIP1;3	Aquaporin TIP1-3	Cluster-29589.56555	O82598.1	3e-107	3H	C4	Korla-specific	Cuticle / water / drought – Aquaporin
ZxTIP2;1	Aquaporin TIP2-1	Cluster-29589.21007	Q41951.2	2e-110	3H	C3	Korla-specific	Cuticle / water / drought – Aquaporin
ZxTIP2;2	Probable aquaporin TIP2-2	Cluster-29589.64171	Q41975.2	6e-115	3H	C4	Korla-specific	Cuticle / water / drought – Aquaporin
ZxGDSL-APG	GDSL esterase/lipase APG	Cluster-29589.19828	Q9LU14.1	4e-141	3H	C2	Wensu-specific	Cuticle / water / drought – Cutin / GDSL
ZxGDSL1	GDSL esterase/lipase At5g03610	Cluster-29589.47812	Q9LZS7.1	1e-111	3H	C4	Korla-specific	Cuticle / water / drought – Cutin / GDSL
ZxGDSL2	GDSL esterase/lipase At5g03610	Cluster-29589.47813	Q9LZS7.1	5e-59	3H	C4	Korla-specific	Cuticle / water / drought – Cutin / GDSL
ZxGDSL3	GDSL esterase/lipase At5g55050	Cluster-29589.2461	Q9FIA1.1	6e-100	3H	C4	Korla-specific	Cuticle / water / drought – Cutin / GDSL
ZxGDSL4	GDSL esterase/lipase At2g42990	Cluster-29589.63707	Q67ZI9.1	3e-113	3H	C6	Leaf-form (specific)	Cuticle / water / drought – Cutin / GDSL

Abbrev.	Best hit description	Gene ID	Accession	E-value	Figure	Cluster	Axis	Classification
ZxCOR47	Dehydrin COR47	Cluster-29589.23211	P31168.2	5e-05	3H	C6	Leaf-form (specific)	Cuticle / water / drought – LEA / dehydrin
ZxDHN1	Dehydrin DHN1	Cluster-29589.27258	Q6ESR4.1	8e-05	3H	C6	Leaf-form (specific)	Cuticle / water / drought – LEA / dehydrin
ZxERD14	dehydrin ERD14_x0001_AGC51777.1 dehydrin protein_x0001_OAY50493.1 hypothetical protein MANES_05G140400	Cluster-29589.64970	NR	1e-04	3H	C4	Leaf-form (graded)	Cuticle / water / drought – LEA / dehydrin
ZxLEA-D29	PREDICTED: late embryogenesis abundant protein D-29 isoform X2	Cluster-26413.0	NR	4e-08	3H	C4	Korla-specific	Cuticle / water / drought – LEA / dehydrin
ZxLEA14A	Late embryogenesis abundant protein Lea14-A	Cluster-29589.58244	P46518.1	1e-57	3H	C4	Korla-specific	Cuticle / water / drought – LEA / dehydrin
ZxGLTP3	Glycolipid transfer protein 3	Cluster-29589.16568	Q9LU33.1	2e-42	3H	C1	Leaf-form (specific)	Cuticle / water / drought – Lipid-transfer protein
ZxLTP1	Non-specific lipid-transfer protein	Cluster-29589.66367	Q43129.1	2e-37	3H	C6	Leaf-form (specific)	Cuticle / water / drought – Lipid-transfer protein
ZxLTP3	Non-specific lipid-transfer protein 3	Cluster-29589.28172	Q43019.1	5e-30	3H	C1	Leaf-form (specific)	Cuticle / water / drought – Lipid-transfer protein
ZxLTPG7	Non-specific lipid transfer protein GPI-anchored 7	Cluster-29589.38514	Q6NLF7.1	2e-15	3H	C4	Korla-specific	Cuticle / water / drought – Lipid-transfer protein
ZxP5CS	Delta-1-pyrroline-5-carboxylate synthase	Cluster-29589.35762	O04015.1	2e-34	3H	C4	Korla-extreme	Cuticle / water / drought – Osmolyte biosynthesis
ZxRFS1	Probable galactinol--sucrose galactosyltransferase 1	Cluster-29589.61063	Q84VX0.1	0e+00	3H	C4	Korla-specific	Cuticle / water / drought – Osmolyte biosynthesis
ZxRFS2	Probable galactinol--sucrose galactosyltransferase 2	Cluster-27659.0	Q94A08.2	0e+00	3H	C4	Korla-specific	Cuticle / water / drought – Osmolyte biosynthesis
ZxFAR1	Fatty acyl-CoA reductase 1	Cluster-29589.8922	Q39152.1	1e-80	3H	C1	Other	Cuticle / water / drought – Wax biosynthesis
ZxKCS10	3-ketoacyl-CoA synthase 10	Cluster-29589.13646	Q570B4.2	3e-248	3H	C5	Wensu-specific	Cuticle / water / drought – Wax biosynthesis
ZxKCS11	3-ketoacyl-CoA synthase 11	Cluster-15957.0	O48780.1	1e-252	3H	C4	Korla-extreme	Cuticle / water / drought – Wax biosynthesis
ZxLACS1	Long chain acyl-CoA synthetase 1	Cluster-29589.22752	O22898.1	7e-273	3H	C3	Korla-specific	Cuticle / water / drought – Wax biosynthesis
ZxLACS6	Long chain acyl-CoA synthetase 6, peroxisomal	Cluster-29589.14262	Q8LPS1.1	1e-295	3H	C6	Leaf-form (specific)	Cuticle / water / drought – Wax biosynthesis
ZxWSD1a	O-acyltransferase WSD1	Cluster-29589.9303	Q93ZR6.1	5e-95	3H	C4	Korla-specific	Cuticle / water / drought – Wax biosynthesis

Abbrev.	Best hit description	Gene ID	Accession	E-value	Figure	Cluster	Axis	Classification
ZxWSD1b	O-acyltransferase WSD1	Cluster-29589.9308	Q93ZR6.1	3e-52	3H	C4	Korla-specific	Cuticle / water / drought – Wax biosynthesis
ZxWSD5	Probable long-chain-alcohol O-fatty-acyltransferase 5	Cluster-29589.7103	Q9FJ76.1	2e-68	3H	C6	Leaf-form (specific)	Cuticle / water / drought – Wax biosynthesis
ZxPetL	Cytochrome b6-f complex subunit 7	Cluster-29589.26086	P80883.1	1e-07	4F	C5	Other	Photosynthesis – Cytochrome b6/f
ZxFd1	Ferredoxin-1, chloroplastic	Cluster-29589.59707	Q43517.1	2e-45	4F	C4	Korla-specific	Photosynthesis – Ferredoxin / NDH
ZxNdhB3	Photosynthetic NDH subunit of subcomplex B 3, chloroplastic	Cluster-29589.21931	Q9LU21.1	1e-29	4F	C3	Other	Photosynthesis – Ferredoxin / NDH
ZxCP29a	Chlorophyll a-b binding protein CP29.3, chloroplastic	Cluster-29589.26983	Q9S7W1.1	7e-42	4F	C4	Korla-specific	Photosynthesis – Light-harvesting antenna
ZxCP29b	Chlorophyll a-b binding protein CP29.3, chloroplastic	Cluster-29589.27621	Q9S7W1.1	9e-42	4F	C4	Korla-specific	Photosynthesis – Light-harvesting antenna
ZxLHCB2	Chlorophyll a-b binding protein of LHCII type I, chloroplastic	Cluster-29589.27018	P08221.1	2e-108	4F	C1	Other	Photosynthesis – Light-harvesting antenna
ZxLHCB3	Chlorophyll a-b binding protein 3C, chloroplastic	Cluster-29589.26985	P07369.1	4e-130	4F	C3	Korla-specific	Photosynthesis – Light-harvesting antenna
ZxLHCB4	Chlorophyll a-b binding protein 3C, chloroplastic	Cluster-29589.27217	P07369.1	4e-109	4F	C3	Korla-specific	Photosynthesis – Light-harvesting antenna
ZxLHCB5	Chlorophyll a-b binding protein 151, chloroplastic	Cluster-29589.27195	P27518.2	2e-123	4F	C5	Leaf-form (specific)	Photosynthesis – Light-harvesting antenna
ZxPsaE	Photosystem I reaction center subunit IV A, chloroplastic	Cluster-29589.39448	Q41228.1	7e-10	4F	C2	Other	Photosynthesis – Photosystem I
ZxPsaK1	Photosystem I reaction center subunit psaK, chloroplastic	Cluster-29589.30387	Q9SUI5.2	3e-31	4F	C5	Leaf-form (graded)	Photosynthesis – Photosystem I
ZxPsaL	Photosystem I reaction center subunit XI, chloroplastic	Cluster-29589.27010	Q41385.1	2e-94	4F	C2	Other	Photosynthesis – Photosystem I
ZxPsaN	Photosystem I reaction center subunit N, chloroplastic	Cluster-29589.28171	P49107.2	8e-52	4F	C5	Wensu-specific	Photosynthesis – Photosystem I
ZxPsaO	Photosystem I subunit O	Cluster-29589.25670	Q949Q5.1	3e-49	4F	C5	Wensu-specific	Photosynthesis – Photosystem I
ZxHCF136	Photosystem II stability/assembly factor HCF136, chloroplastic	Cluster-29589.24859	O82660.1	6e-144	4F	C3	Korla-specific	Photosynthesis – Photosystem II
ZxPsb28	Photosystem II reaction center PSB28 protein, chloroplastic	Cluster-29589.29692	Q0JG75.2	5e-57	4F	C1	Leaf-form (specific)	Photosynthesis – Photosystem II
ZxPsbC	Photosystem II CP43 reaction center protein	Cluster-29589.47825	B1A931.1	2e-282	4F	C3	Korla-specific	Photosynthesis – Photosystem II
ZxPsbO1	Oxygen-evolving enhancer protein 1, chloroplastic	Cluster-29589.24817	P23322.2	3e-130	4F	C2	Leaf-form (specific)	Photosynthesis – Photosystem II

Abbrev.	Best hit description	Gene ID	Accession	E-value	Figure	Cluster	Axis	Classification
ZxPsbO2	Oxygen-evolving enhancer protein 2, chloroplastic	Cluster-29589.27701	P93566.1	8e-89	4F	C1	Leaf-form (specific)	Photosynthesis – Photosystem II
ZxPsbS	Photosystem II 22 kDa protein, chloroplastic	Cluster-29589.30456	Q9XF91.1	4e-88	4F	C6	Other	Photosynthesis – Photosystem II
ZxPGKc2	Phosphoglycerate kinase, cytosolic	Cluster-29589.23207	Q42962.1	4e-206	4G	C2	Wensu-specific	Carbon metabolism – cytosolic phosphoglycerate kinase
ZxCA1	Carbonic anhydrase, chloroplastic	Cluster-29589.30876	P16016.2	9e-112	4G	C3	Korla-specific	Photosynthesis – Calvin-cycle carbon fixation
ZxCA2	Carbonic anhydrase, chloroplastic	Cluster-29589.27942	P27141.1	2e-67	4G	C3	Korla-specific	Photosynthesis – Calvin-cycle carbon fixation
ZxRBCL	Ribulose bisphosphate carboxylase large chain	Cluster-29589.31028	Q32223.1	2e-05	4G	C3	Korla-specific	Photosynthesis – Calvin-cycle carbon fixation
ZxRBCS1	Ribulose bisphosphate carboxylase small chain SSU26, chloroplastic	Cluster-29589.26900	P19308.1	2e-15	4G	C3	Korla-specific	Photosynthesis – Calvin-cycle carbon fixation
ZxRBCS2	Ribulose bisphosphate carboxylase small chain, chloroplastic	Cluster-29589.27005	P24007.1	1e-55	4G	C3	Korla-specific	Photosynthesis – Calvin-cycle carbon fixation
ZxRCA1	Ribulose bisphosphate carboxylase/oxygenase activase 1, chloroplastic	Cluster-29589.2227	Q7X9A0.1	8e-222	4G	C4	Korla-specific	Photosynthesis – Calvin-cycle carbon fixation
ZxCHLH1	Magnesium-chelatase subunit ChlH, chloroplastic	Cluster-29589.27313	Q9FNB0.1	0e+00	4G	C3	Korla-specific	Photosynthesis – Chlorophyll biosynthesis
ZxCHLH2	Magnesium-chelatase subunit ChlH, chloroplastic	Cluster-29589.27326	Q9FNB0.1	2e-76	4G	C3	Korla-specific	Photosynthesis – Chlorophyll biosynthesis
ZxCHLH3	Magnesium-chelatase subunit ChlH, chloroplastic	Cluster-29589.24929	Q9FNB0.1	0e+00	4G	C3	Korla-specific	Photosynthesis – Chlorophyll biosynthesis
ZxPOR1	Protochlorophyllide reductase, chloroplastic	Cluster-29589.30426	Q41249.1	2e-171	4G	C3	Korla-specific	Photosynthesis – Chlorophyll biosynthesis
ZxPOR2	Protochlorophyllide reductase, chloroplastic	Cluster-29589.24579	Q01289.1	7e-170	4G	C1	Leaf-form (specific)	Photosynthesis – Chlorophyll biosynthesis
ZxPTC52	Protochlorophyllide-dependent translocon component 52, chloroplastic	Cluster-29589.34065	Q8W496.1	1e-66	4G	C4	Korla-specific	Photosynthesis – Chlorophyll biosynthesis
ZxCURT1B	Protein CURVATURE THYLAKOID 1B, chloroplastic	Cluster-29589.26997	Q8LCA1.2	2e-07	4G	C1	Leaf-form (specific)	Photosynthesis – Thylakoid organisation
ZxPlsp1	Thylakoidal processing peptidase 1, chloroplastic	Cluster-29589.44843	O04348.2	4e-80	4G	C2	Other	Photosynthesis – Thylakoid organisation
ZxTAAC	Thylakoid ADP,ATP carrier protein, chloroplastic	Cluster-29589.19657	Q9M024.1	7e-65	4G	C3	Korla-specific	Photosynthesis – Thylakoid organisation
ZxTL17.9	Thylakoid lumenal 17.9 kDa protein,	Cluster-29589.59242	Q9SW33.1	1e-63	4G	C3	Korla-specific	Photosynthesis – Thylakoid organisation

Abbrev.	Best hit description	Gene ID	Accession	E-value	Figure	Cluster	Axis	Classification
	chloroplastic							
ZxCP1	Calcium-binding protein CP1	Cluster-29589.43057	Q9FDX6.1	4e-36	5	C4	Korla-extreme	Site-associated stress response
ZxMYB4	Transcription factor MYB4	Cluster-23309.2	Q7XBH4.2	6e-60	5	C6	Leaf-form (graded)	Site-associated stress response
ZxPDR1	Pleiotropic drug resistance protein 1	Cluster-29589.56456	Q76CU2.1	0e+00	5	C4	Korla-extreme	Site-associated stress response
ZxPR4	Pathogenesis-related protein PR-4A	Cluster-29589.31587	P29062.1	5e-54	5	C4	Korla-extreme	Site-associated stress response
ZxPGKc	Phosphoglycerate kinase, cytosolic	Cluster-29589.23198	Q42962.1	3e-206	4G, 4D	C2	Leaf-form (graded)	Carbon metabolism – cytosolic phosphoglycerate kinase
ZxCAB	Chlorophyll a-b binding protein P4, chloroplastic	Cluster-29589.27918	Q9SQL2.1	2e-126	4F, 4D	C1	Leaf-form (graded)	Photosynthesis – Light-harvesting antenna
ZxLHCB1	Chlorophyll a-b binding protein 3C, chloroplastic	Cluster-29589.27121	P07369.1	4e-139	4F, 4D	C2	Leaf-form (graded)	Photosynthesis – Light-harvesting antenna

Gene abbreviations denote the best DIAMOND/BLAST hit in Swiss-Prot or NR and indicate putative homology only; orthology was not established phylogenetically. "Cluster" is the k-means co-expression cluster (C1–C6) for genes for which normalised expression estimates were available for all three accessions (—, not in that set). "Axis" gives the assignment defined in Section 2.6. Leaf-form (specific) and Leaf-form (graded) are the two classes of the leaf-form–associated set: both are differentially expressed concordantly in the two flat-versus-rod comparisons, so that the flat-leaved accession lies at one extreme of the series, and they differ only in whether the two rod-leaved accessions also differ from each other (graded) or not (specific). Korla-specific and Wensu-specific denote genes that set one rod-leaved accession apart from the other two. Korla-extreme denotes genes differentially expressed in all three comparisons whose direction reverses between the two flat-versus-rod comparisons, so that Korla lies at one extreme and Guide is intermediate. Other denotes DEGs that fall into none of these classes.

Table S8. Size, mean expression profile and marker genes of the six co-expression clusters.

Cluster	Genes	Guide	Korla	Wensu	Representative marker genes
C1	620	1.37 ± 0.05	−0.78 ± 0.27	−0.58 ± 0.31	LHCII-type CAB, CP12-2, ribulose-5-phosphate-3-epimerase, catalase 1
C2	517	0.97 ± 0.29	0.35 ± 0.37	−1.31 ± 0.09	chlorophyll a-b binding protein 3C, triose-phosphate translocator, PEPC4
C3	488	0.62 ± 0.41	−1.34 ± 0.10	0.72 ± 0.35	RuBisCO small chain, magnesium-chelatase subunit ChlH, dicarboxylate transporter 1
C4	1,282	−0.49 ± 0.35	1.35 ± 0.09	−0.86 ± 0.28	PR-1, endochitinase EP3, thaumatin-like protein 1, ACC oxidase 3
C5	515	−1.10 ± 0.30	−0.11 ± 0.45	1.21 ± 0.17	photosystem I subunit N, glutamine synthetase, glutathione S-transferase U17
C6	763	−1.32 ± 0.10	0.97 ± 0.25	0.35 ± 0.34	RD21B cysteine protease, HMG-CoA synthase, 1-deoxy-D-xylulose-5-phosphate synthase

Clusters are those of Figure 3C and 3F. Guide, Korla and Wensu give the mean row z-score of the cluster ± the standard deviation across its genes; the row z-score standardises the three accession means of each gene to a mean of 0 and a standard deviation of 1, so the values describe the shape of the expression profile and not its absolute level. Marker genes are the annotations of representative members of each cluster and are the genes previously listed beneath the panels of Figure 3F.

Table S9. Expression of the actin reference gene in the three accessions.

Accession (collection no.)	Replicate 1	Replicate 2	Replicate 3	Mean ± SD	CV within accession (%)
Guide (Chensl1200)	555.82	572.78	557.56	562.05 ± 9.33	1.66
Korla (LiZJ1402)	438.16	445.81	474.18	452.72 ± 18.98	4.19
Wensu (LiZJ0039)	512.48	500.46	498.95	503.96 ± 7.41	1.47

Values are transcripts per million (TPM) for Cluster-29589.26477, the most abundant near-full-length actin transcript of the assembly (1,837 bp; best Swiss-Prot hit Actin-7, P53492, *Arabidopsis thaliana*), in the three biological replicates of each accession; CV is the coefficient of variation among the three replicates of that accession. The highest and the lowest accession means differ 1.24-fold, and the coefficient of variation among the three accession means is 10.8 %. The residual difference among accessions is detected by one-way analysis of variance ($F = 53.6$, $P = 1.5 \times 10−4$) because dispersion among replicates is very low; expressed in Ct, a 1.24-fold range corresponds to about 0.3 cycles, against the 2.7- to 36-fold changes of the target genes assayed by qPCR (Table S6). Actin expression is therefore stable enough for normalisation across the three accessions.

Table S10. Gene-specific primers.

Gene	Annotation	Forward primer (5′→3′)	Reverse primer (5′→3′)
ZxPDR1	Pleiotropic drug resistance protein 1 (ABCG/PDR transporter)	CCGGTGTCCACAGTATTCCTG	TTGCCCTCTGATGTCGATGCA
ZxPR4	Pathogenesis-related protein PR-4	GCTTCTGCCAGTGCCCAAA	ACAAGCATCTTTGCCATGAGGT
ZxCP1	Calcium-binding protein CP1	GGTCGATGATATCTGTAGCGGACT	GCCGCCGTTGTATTCACCAC
ZxMYB4	Transcription factor MYB4	GCCATGCTCGAACTATCGGC	CAGAATTCTGCATCCCCAACGT
ZxACT	Actin (reference gene)	CGTGTTGCTCCCGAGGAGCA	TGGCATGAGGGAGCGCATAC

Supplementary Figures

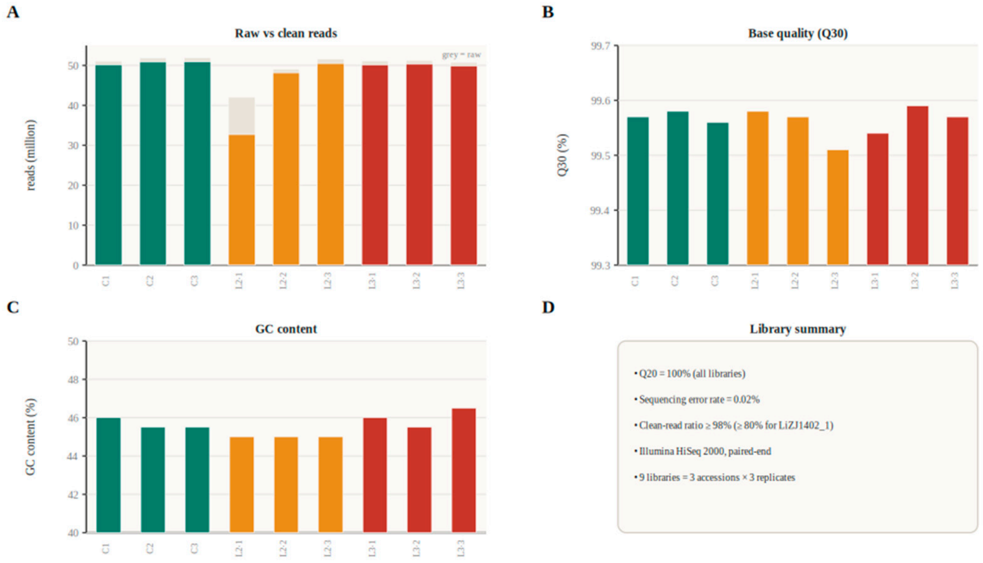

Figure S1. Sequencing output and read quality for the nine leaf libraries. (A) Raw (grey) and clean (coloured) read counts per library; the grey margin marks the reads removed by quality filtering, which was appreciable only for the first Korla replicate. (B) Percentage of bases with a quality score of at least Q30. (C) GC content. (D) Summary of library and platform statistics. Libraries are labelled by accession and replicate: C1–C3, Guide (collection Chensl1200); L2:1–L2:3, Korla (LiZJ1402); L3:1–L3:3, Wensu (LiZJ0039). Per-library values are given in Table S1.

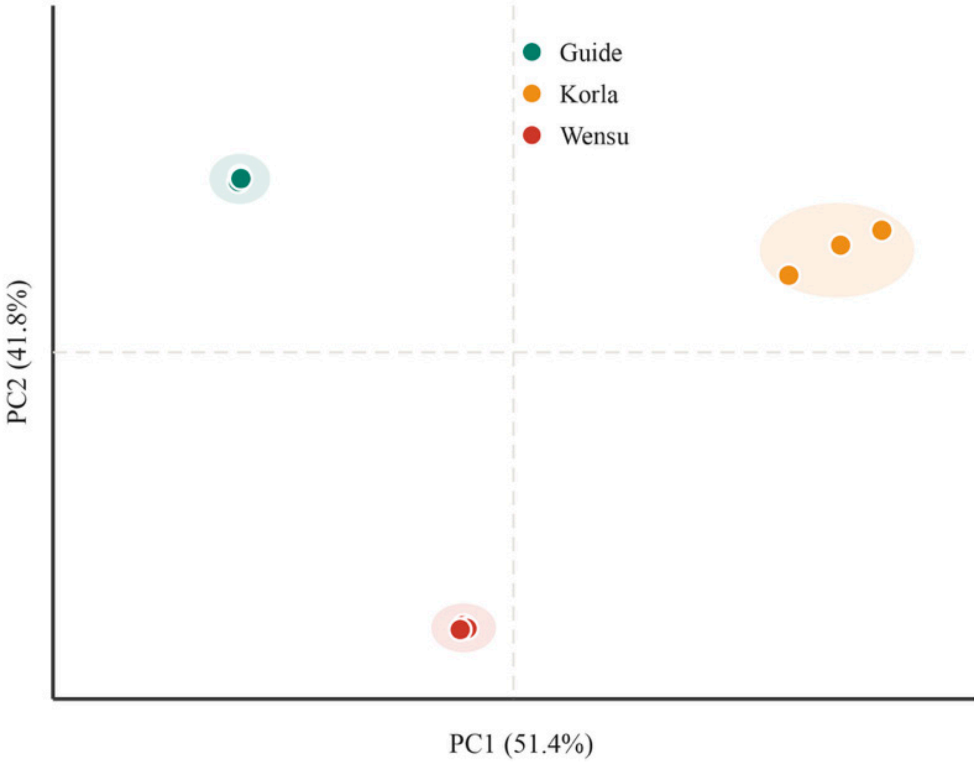

Figure S2. Principal component analysis of the nine leaf transcriptomes. Samples are plotted on the first two principal components of the log-transformed normalised counts; shaded ellipses enclose the three replicates of each accession. PC1, which accounts for 51.4 % of the variance, separates Korla from Guide, with Wensu intermediate; PC2 (41.8 %) separates Wensu from the other two accessions. Replicates of each accession cluster tightly relative to the distances between accessions, indicating that between-accession differences greatly exceed the variation among replicate libraries.

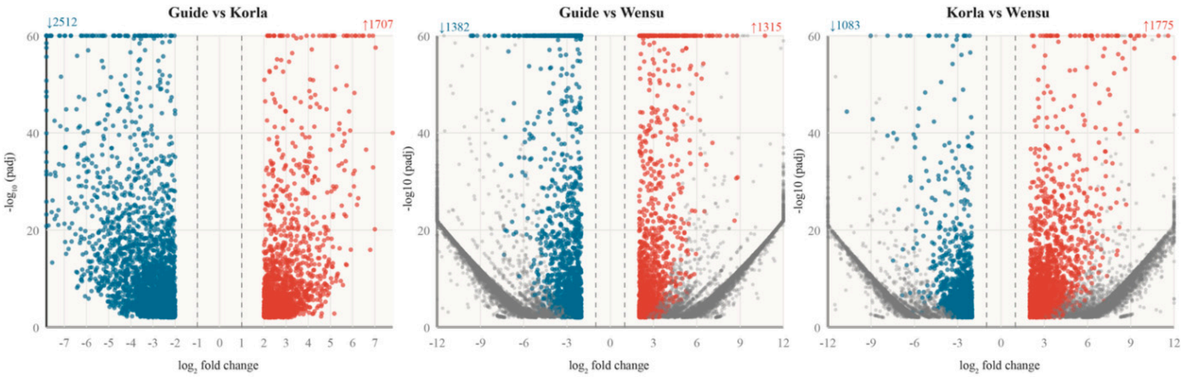

Figure S3. Volcano plots of the three pairwise comparisons. Left, Guide versus Korla; centre, Guide versus Wensu; right, Korla versus Wensu. Each point is a gene, plotted as $\log_2$ fold change against —$\log_{10}$ adjusted *P*.

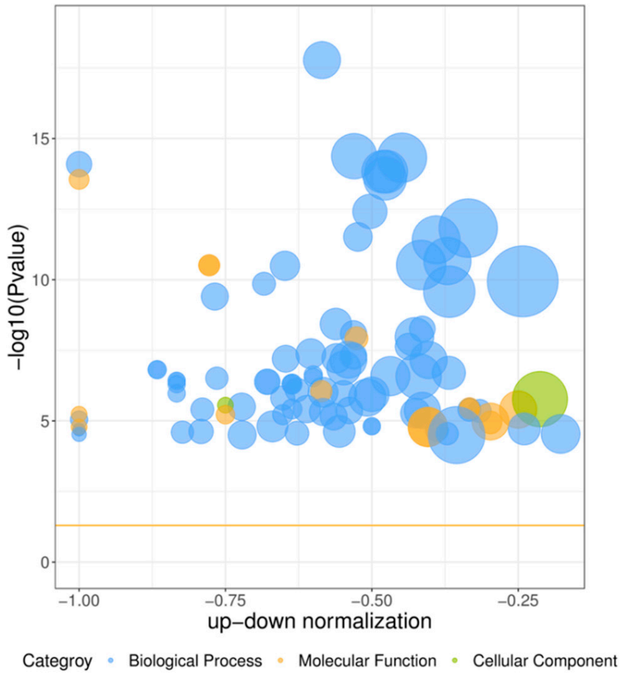

GO term Top 20

ID	Descrption
GO:0042493	response to drug
GO:0001101	response to acid chemical
GO:1901700	response to oxygen−containing compound
GO:0010200	response to chitin
GO:0043207	response to external biotic stimulus
GO:0051707	response to other organism
GO:0009607	response to biotic stimulus
GO:0080046	quercetin 4'−O−glucosyltransferase activity
GO:0009636	response to toxic substance
GO:0042221	response to chemical
GO:0006720	isoprenoid metabolic process
GO:0009725	response to hormone
GO:0009605	response to external stimulus
GO:0009719	response to endogenous stimulus
GO:0009753	response to jasmonic acid
GO:0080043	quercetin 3−O−glucosyltransferase activity
GO:0080044	quercetin 7−O−glucosyltransferase activity
GO:0050896	response to stimulus
GO:0009627	systemic acquired resistance
GO:0010033	response to organic substance

Figure S4. Gene Ontology enrichment, Guide versus Korla. Each bubble is an enriched GO term, plotted as the normalised balance between up- and down-regulated genes within the term (x axis) against $-\log_{10} P$ (y axis); bubble area is proportional to the number of differentially expressed genes annotated to the term and colour denotes ontology. The horizontal line marks $P = 0.05$. The accompanying table lists the twenty most significantly enriched terms. Terms describing responses to chemical, biotic and hormonal stimuli dominate, together with three quercetin glucosyltransferase activities, consistent with the secondary-metabolism enrichment reported for the Korla-specific gene set.

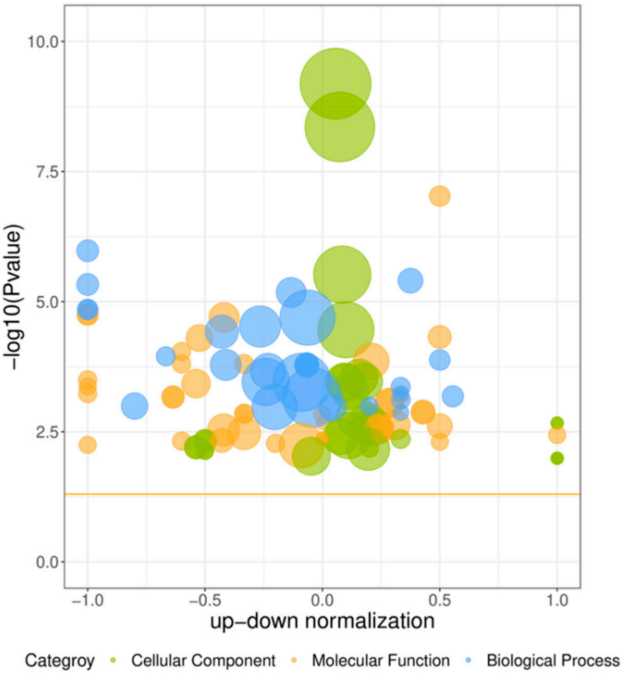

GO term Top 20

ID	Descrption
GO:0009536	plastid
GO:0009507	chloroplast
GO:0004315	3−oxoacyl−[acyl−carrier−protein] synthase activity
GO:0044435	plastid part
GO:0016104	triterpenoid biosynthetic process
GO:0044434	chloroplast part
GO:0009631	cold acclimation
GO:0006722	triterpenoid metabolic process
GO:0031559	oxidosqualene cyclase activity
GO:0080046	quercetin 4'−O−glucosyltransferase activity
GO:0008194	UDP−glycosyltransferase activity
GO:0009624	response to nematode
GO:0019742	pentacyclic triterpenoid metabolic process
GO:0019745	pentacyclic triterpenoid biosynthetic process
GO:0004312	fatty acid synthase activity
GO:0035251	UDP−glucosyltransferase activity
GO:0001101	response to acid chemical
GO:0008610	lipid biosynthetic process
GO:0006720	isoprenoid metabolic process
GO:0004373	glycogen (starch) synthase activity

Figure S5. Gene Ontology enrichment, Guide versus Wensu. Plotted as in Figure S4. The most significantly enriched terms in this comparison are the plastid and chloroplast cellular components, the only comparison in which photosynthesis-related terms head the list; terpenoid and lipid metabolic processes and glycosyltransferase activities follow.

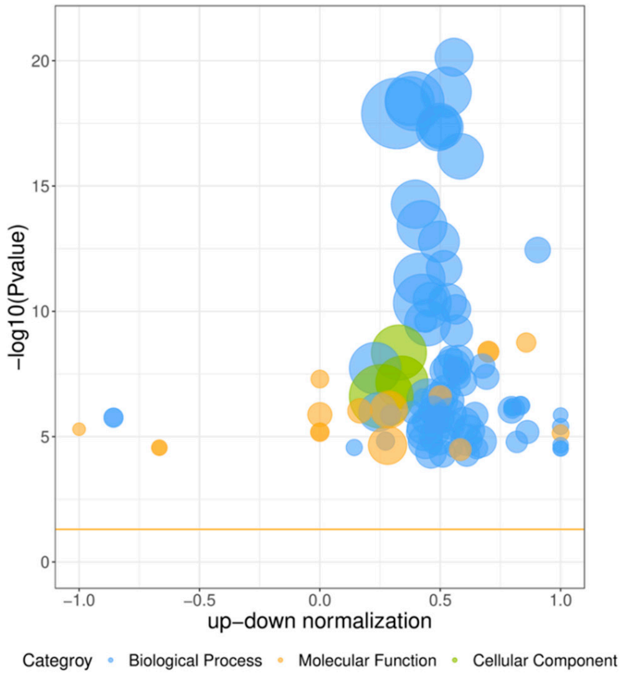

GO term Top 20

ID	Descrption
GO:0042493	response to drug
GO:1901700	response to oxygen-containing compound
GO:0009605	response to external stimulus
GO:0042221	response to chemical
GO:0050896	response to stimulus
GO:0043207	response to external biotic stimulus
GO:0051707	response to other organism
GO:0009607	response to biotic stimulus
GO:0001101	response to acid chemical
GO:0009725	response to hormone
GO:0009719	response to endogenous stimulus
GO:0006952	defense response
GO:0010200	response to chitin
GO:0009617	response to bacterium
GO:0010033	response to organic substance
GO:0009636	response to toxic substance
GO:0098542	defense response to other organism
GO:0006950	response to stress
GO:0009753	response to jasmonic acid
GO:1900055	regulation of leaf senescence

Figure S6. Gene Ontology enrichment, Korla versus Wensu. Plotted as in Figure S4. Enrichment in this comparison is almost entirely within the biological-process ontology and is dominated by terms describing responses to chemical, external and biotic stimuli, defence responses and hormone signalling.

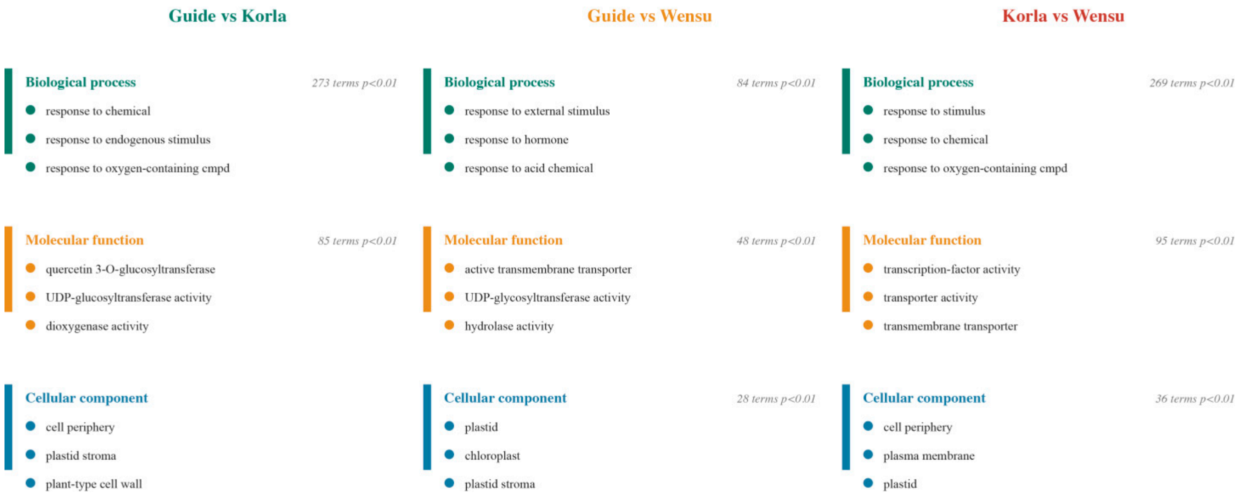

Figure S7. The three most significantly enriched Gene Ontology terms per ontology in each comparison. Terms are grouped by ontology (biological process, molecular function, cellular component) and ranked by *P* value; the number of terms significant at *P* < 0.01 is given at the right of each block.

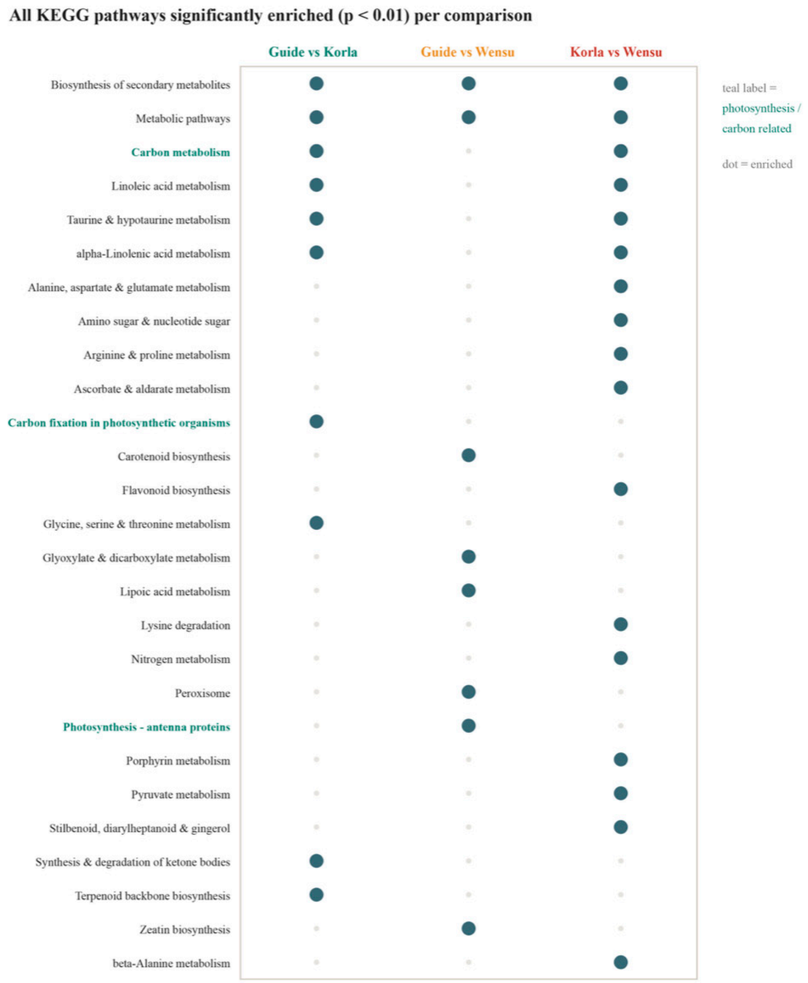

Figure S8. KEGG pathways significantly enriched in at least one comparison. Filled circles mark the comparisons in which a pathway was significantly enriched (*P* < 0.01); small open dots mark comparisons in which it was not. Pathways related to photosynthesis and carbon metabolism are labelled in teal. Comparison codes are as in Figure S7. Biosynthesis of secondary metabolites and general metabolic pathways are enriched in all three comparisons, whereas carbon fixation in photosynthetic organisms and photosynthesis antenna proteins are enriched only in comparisons involving the flat-leaved Guide accession. Complete pathway lists are given in Table S4.
